# Supplementary material for: Conserved genetic markers reveal widespread diatom sexual reproduction in the global ocean
Source: Nat Commun. 2025 Nov 14;16:10029. doi: 10.1038/s41467-025-65296-9 (PMC12618581; doi:10.1038/s41467-025-65296-9)
Supplement: Supplementary file 1 — Supplementary Information [file 41467_2025_65296_MOESM1_ESM.pdf]

Supplementary Information: Conserved genetic markers reveal widespread diatom sexual reproduction in the global ocean

Gust Bilcke[x1,2,3], Lucia Campese[x4], Rossella Annunziata[4], Luz Amadei Martínez[3], Camilla Borgonuovo[4], Nadine Rijsdijk[1,2,3], Peter Chaerle[3,5], Koen Van den Berge[6], Sofie D'hondt[3], Daniele Iudicone[4], Marina Montresor[4], Maria Immacolata Ferrante[+*4,7], Klaas Vandepoele[+*1,2,8], Wim Vyverman[+*3]

1. Department of Plant Biotechnology and Bioinformatics, Ghent University, Technologiepark 71, 9052 Ghent, Belgium
2. VIB Center for Plant Systems Biology, Technologiepark 71, 9052 Ghent, Belgium
3. Protistology and Aquatic Ecology, Department of Biology, Ghent University, 9000, Ghent, Belgium
4. Stazione Zoologica Anton Dohrn, 80121 Napoli, Italy.
5. BCCM/DCG Diatoms Collection, Department of Biology, Ghent University, 9000 Ghent, Belgium
6. Statistics and Decision Sciences, Johnson and Johnson, Beerse, Belgium
7. National Institute of Oceanography and Applied Geophysics, Trieste, Italy
8. VIB Center for AI & Computational Biology, VIB, Ghent, Belgium

[x]These authors contributed equally: Gust Bilcke, Lucia Campese

[+]These authors jointly supervised this work: Maria Immacolata Ferrante, Klaas Vandepoele, Wim Vyverman

* **Corresponding authors**:
Mariella.Ferrante@szn.it
Klaas.Vandepoele@psb.ugent.be
Wim.Vyverman@UGent.be

Supplementary Figures

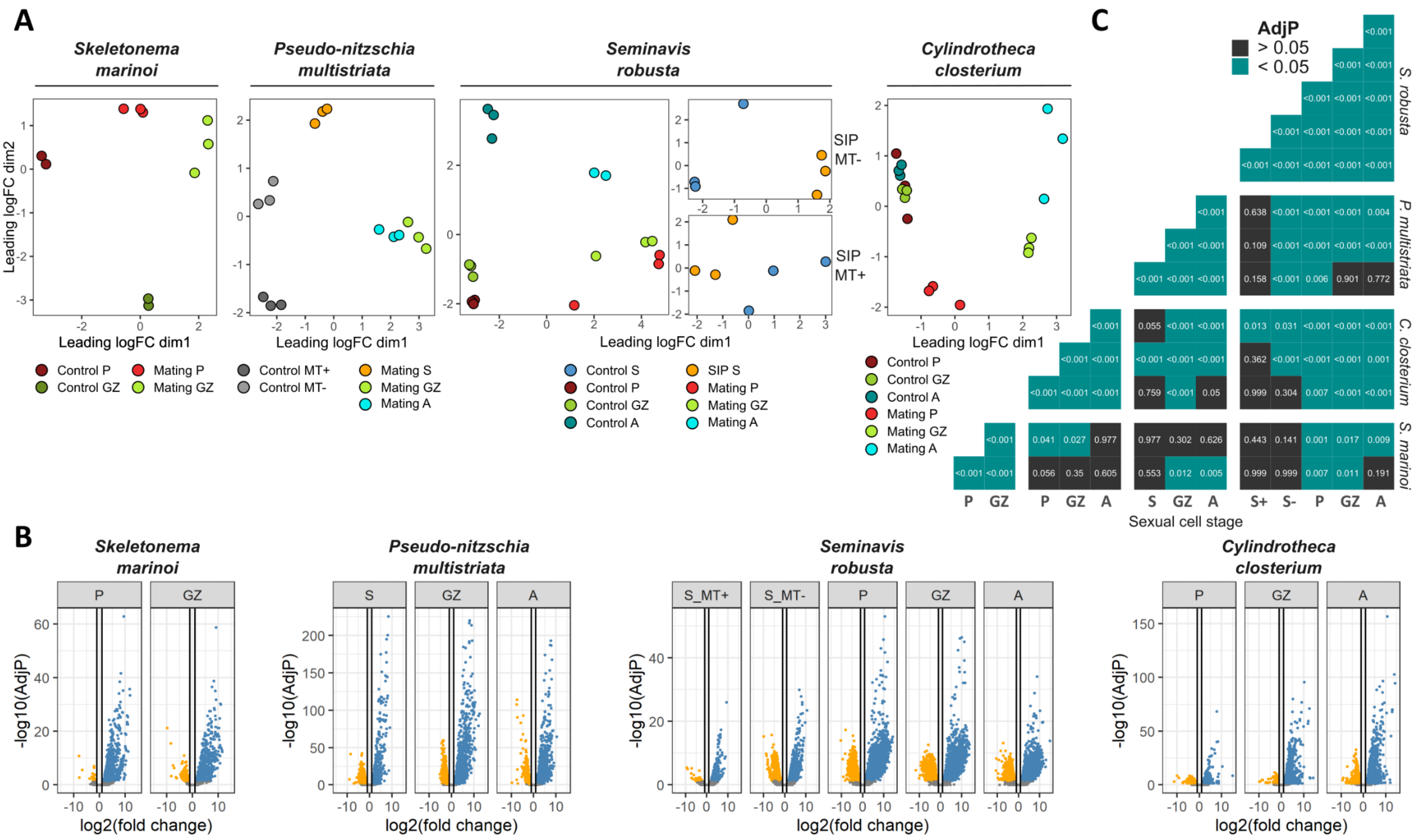
A
Skeletonema marinoi
Pseudo-nitzschia multistriata
Seminavis robusta
Cylindrotheca closterium
Leading logFC dim1
Leading logFC dim2
Control P
Mating P
Control GZ
Mating GZ
Control MT+
Control MT-
Mating S
Mating A
Control S
Control A
SIP S
SIP MT-
SIP MT+
B
log2(fold change)
-log10(AdjP)
S_MT+
S_MT-
C
AdjP
> 0.05
< 0.05
S. robusta
P. multistriata
C. closterium
S. marinoi
Sexual cell stage

Supplementary Figure 1: **Differential expression analyses for four diatom species undergoing sexual reproduction**. **(A)** Multidimensional scaling (MDS) scatterplots. Points represent individual transcriptomic samples, which are coloured according to treatment (control or mating) and time point (S: pheromone signaling, P: gametangia, pre-gametic, GZ: gametes and zygotes, A: auxospores). MT: mating type, SIP: sex inducing pheromone **(B)** Volcano plots showing -log10(adjusted p-value) in function of the log2(fold change) for each contrast of each species. Significant genes are coloured in blue (upregulated during sex) and orange (downregulated during sex). **(C)** Matrix showing the adjusted p-values from paired association tests between the log2 fold changes of differentially expressed genes across different sexual cell stages and species (see associated Pearson correlation coefficients in Figure 1c), adjusted using the Benjamini-Hochberg procedure.

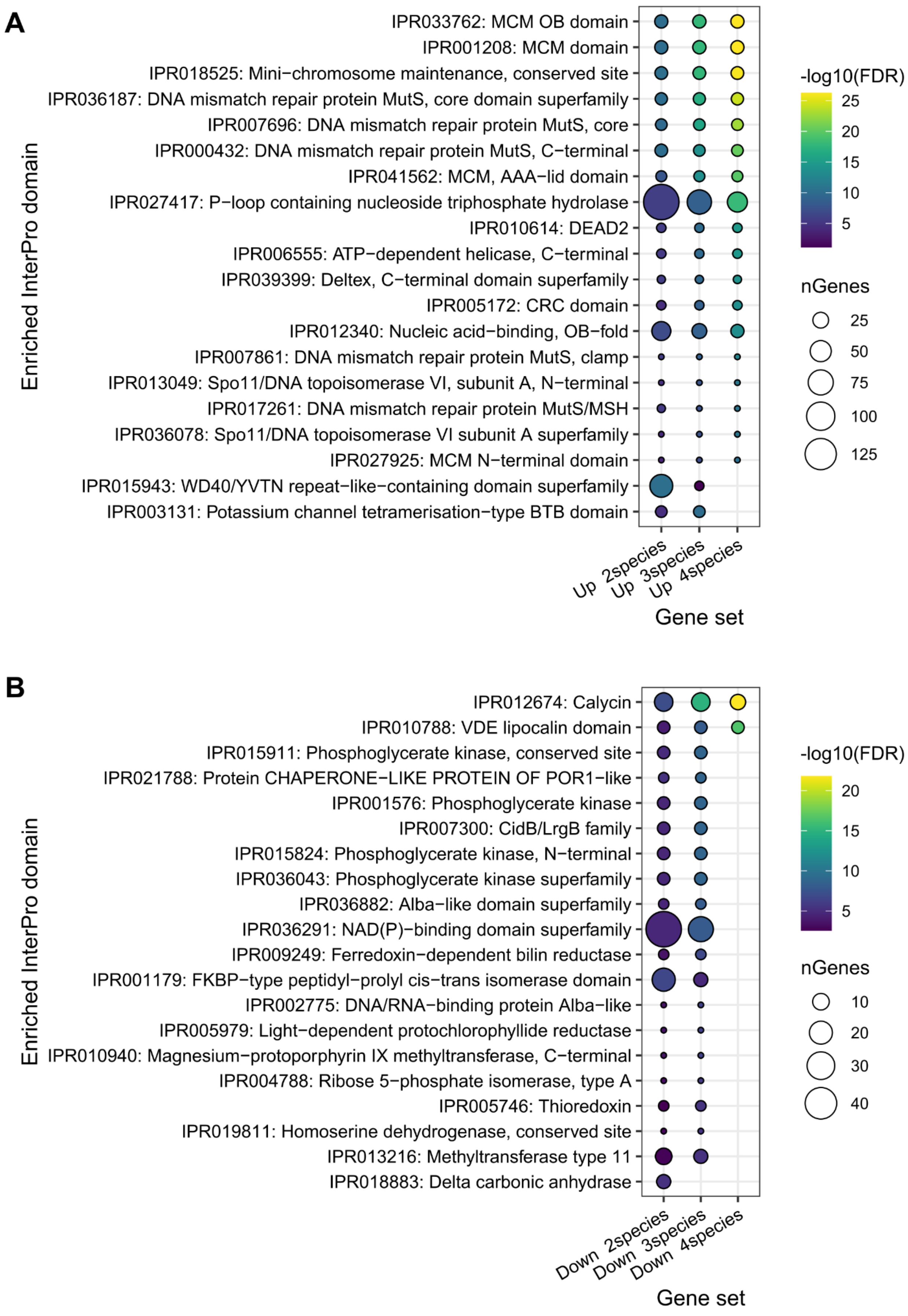
A
Enriched InterPro domain
IPR033762: MCM OB domain
IPR001208: MCM domain
IPR018525: Mini−chromosome maintenance, conserved site
IPR036187: DNA mismatch repair protein MutS, core domain superfamily
IPR007696: DNA mismatch repair protein MutS, core
IPR000432: DNA mismatch repair protein MutS, C−terminal
IPR041562: MCM, AAA−lid domain
IPR027417: P−loop containing nucleoside triphosphate hydrolase
IPR010614: DEAD2
IPR006555: ATP−dependent helicase, C−terminal
IPR039399: Deltex, C−terminal domain superfamily
IPR005172: CRC domain
IPR012340: Nucleic acid−binding, OB−fold
IPR007861: DNA mismatch repair protein MutS, clamp
IPR013049: Spo11/DNA topoisomerase VI, subunit A, N−terminal
IPR017261: DNA mismatch repair protein MutS/MSH
IPR036078: Spo11/DNA topoisomerase VI subunit A superfamily
IPR027925: MCM N−terminal domain
IPR015943: WD40/YVTN repeat−like−containing domain superfamily
IPR003131: Potassium channel tetramerisation−type BTB domain
Up 2species
Up 3species
Up 4species
Gene set
-log10(FDR)
25
20
15
10
5
nGenes
25
50
75
100
125
B
Enriched InterPro domain
IPR012674: Calycin
IPR010788: VDE lipocalin domain
IPR015911: Phosphoglycerate kinase, conserved site
IPR021788: Protein CHAPERONE−LIKE PROTEIN OF POR1−like
IPR001576: Phosphoglycerate kinase
IPR007300: CidB/LrgB family
IPR015824: Phosphoglycerate kinase, N−terminal
IPR036043: Phosphoglycerate kinase superfamily
IPR036882: Alba−like domain superfamily
IPR036291: NAD(P)−binding domain superfamily
IPR009249: Ferredoxin−dependent bilin reductase
IPR001179: FKBP−type peptidyl−prolyl cis−trans isomerase domain
IPR002775: DNA/RNA−binding protein Alba−like
IPR005979: Light−dependent protochlorophyllide reductase
IPR010940: Magnesium−protoporphyrin IX methyltransferase, C−terminal
IPR004788: Ribose 5−phosphate isomerase, type A
IPR005746: Thioredoxin
IPR019811: Homoserine dehydrogenase, conserved site
IPR013216: Methyltransferase type 11
IPR018883: Delta carbonic anhydrase
Down 2species
Down 3species
Down 4species
Gene set
-log10(FDR)
20
15
10
5
nGenes
10
20
30
40

Supplementary Figure 2: Bubble plots showing the top-20 most significantly enriched InterPro domains (y-axis) of differentially expressed genes from families with a shared response in 2, 3 and 4 species (x-axis). Statistical testing of enrichment was performed with the hypergeometric test from the clusterProfiler package for R [1], controlling the false discovery rate (FDR) at 0.05 **(A)** Enrichment within shared gene families consisting of only upregulated genes during sex. **(B)** Enrichment within shared gene families consisting of only downregulated genes during sex.

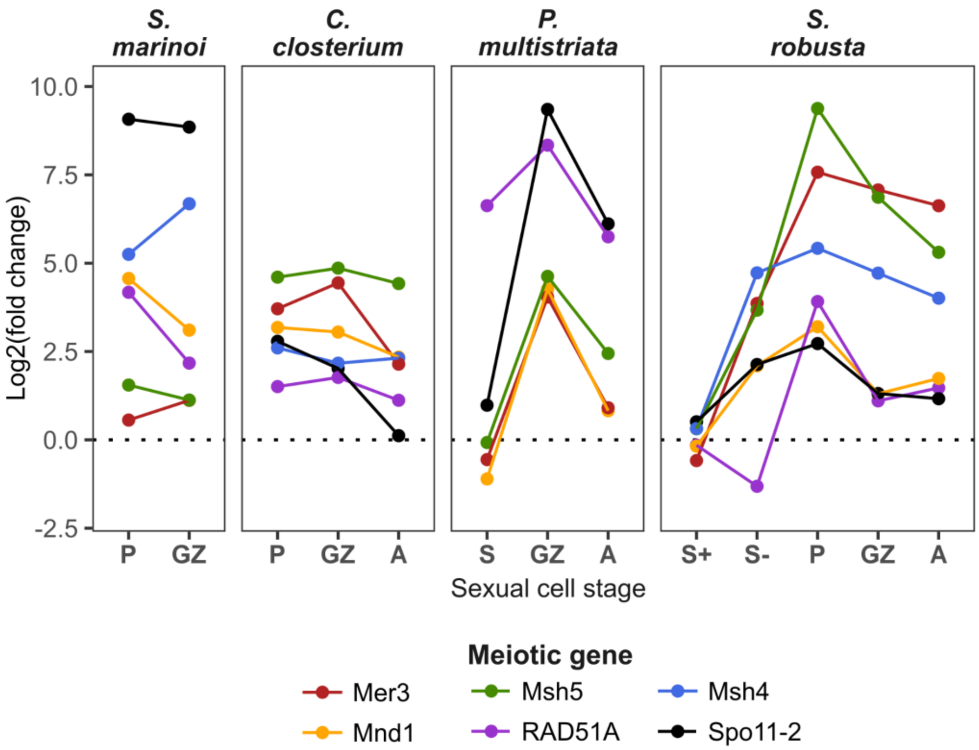

Supplementary Figure 3: Line plots showing log2 fold changes of meiotic genes throughout different sexual cell stages in four sexual species. S: pheromone signaling, P: gametangia, pre-gametic, GZ: gametes and zygotes, A: auxospores.

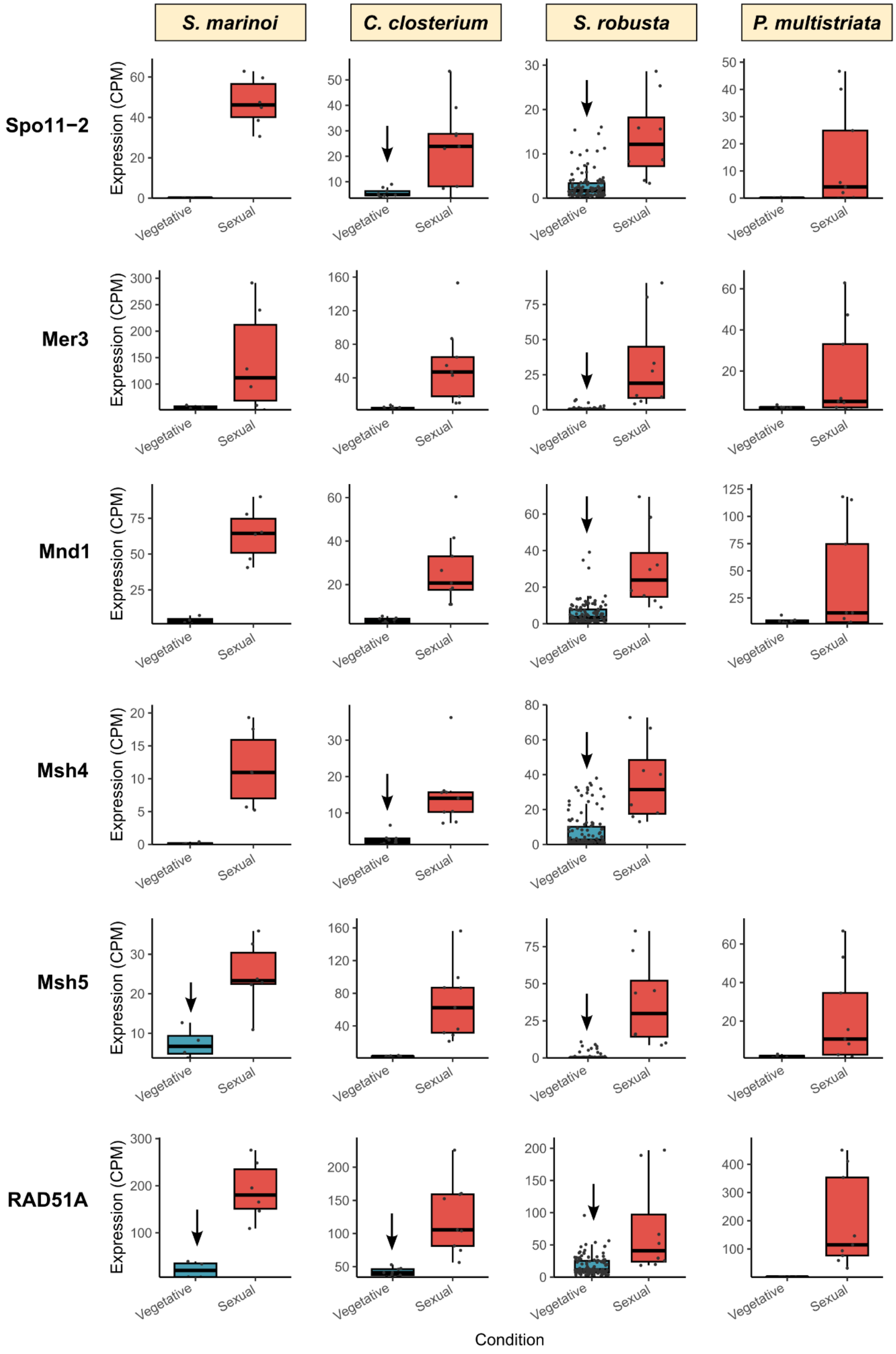

S. marinoi
C. closterium
S. robusta
P. multistriata
Spo11−2
Mer3
Mnd1
Msh4
Msh5
RAD51A
Expression (CPM)
Vegetative
Sexual
Condition

Supplementary Figure 4: Box plots comparing expression levels (in counts per million) for six meiotic genes in vegetative conditions (blue) versus sexual reproduction (red). For *S. robusta*, all vegetative samples of the *S. robusta* expression atlas are included as “vegetative”. Individual data points are shown as dots. Arrows point out expression in vegetative conditions. The central line of the boxplot indicates the median, the box limits show the 25th and 75th percentiles and whiskers extend up to 1.5x the interquartile range.

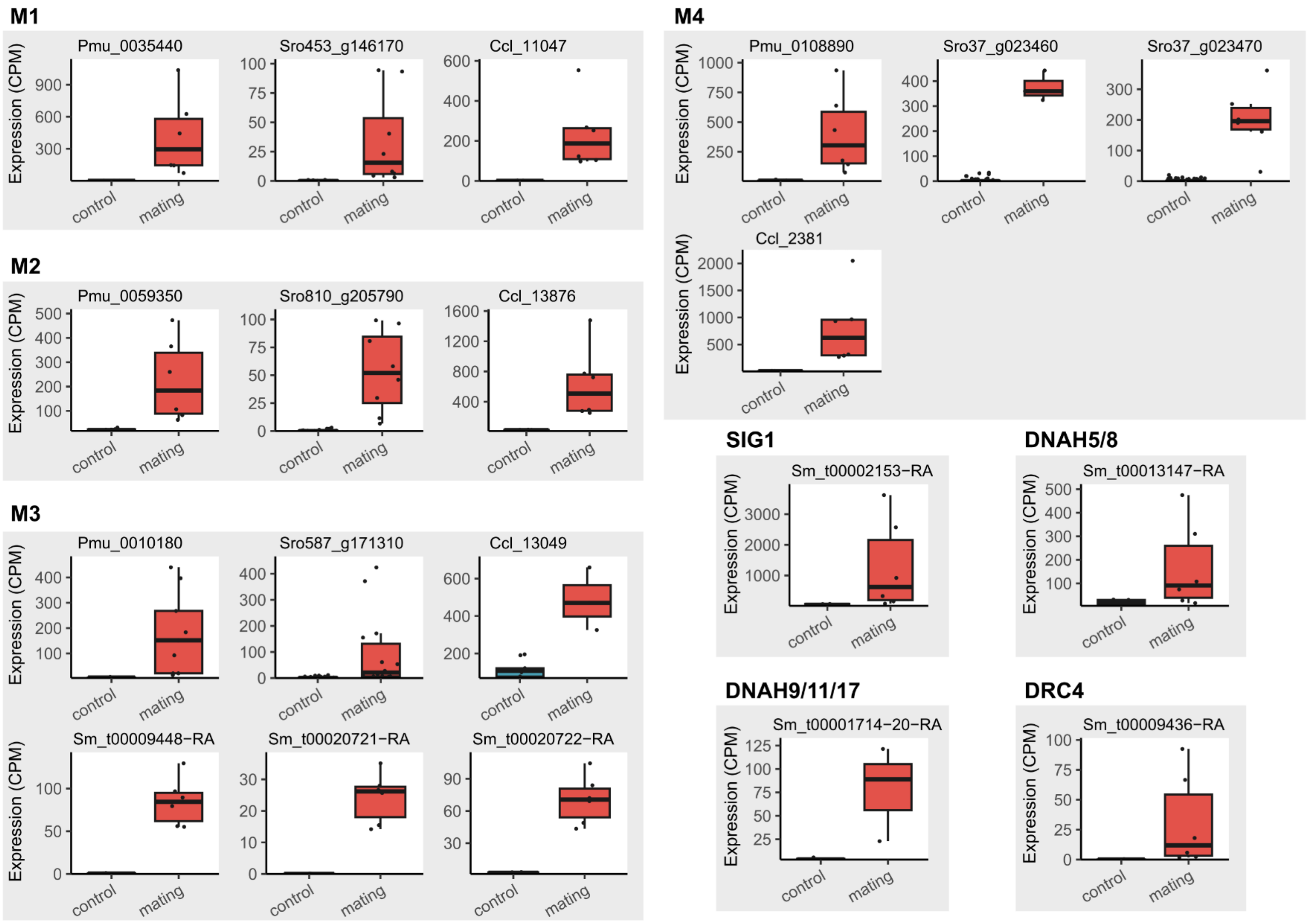

Supplementary Figure 5: Box plots showing the expression in counts per million (CPM) of a panel of eight sex marker families in four diatom species (Pmu: *Pseudo-nitzschia multistriata*, Sro: *Seminavis robusta*, Ccl: *Cylindrotheca closterium* and Sm: *Skeletonema marinoi*). Expression is shown in vegetative (control, blue) and upregulated sexual (mating, red) conditions. For *S. robusta*, all vegetative samples of the *S. robusta* expression atlas are included as “vegetative”. Dots show individual replicates. The central line of the boxplot indicates the median, the box limits show the 25th and 75th percentiles and whiskers extend up to 1.5x the interquartile range.

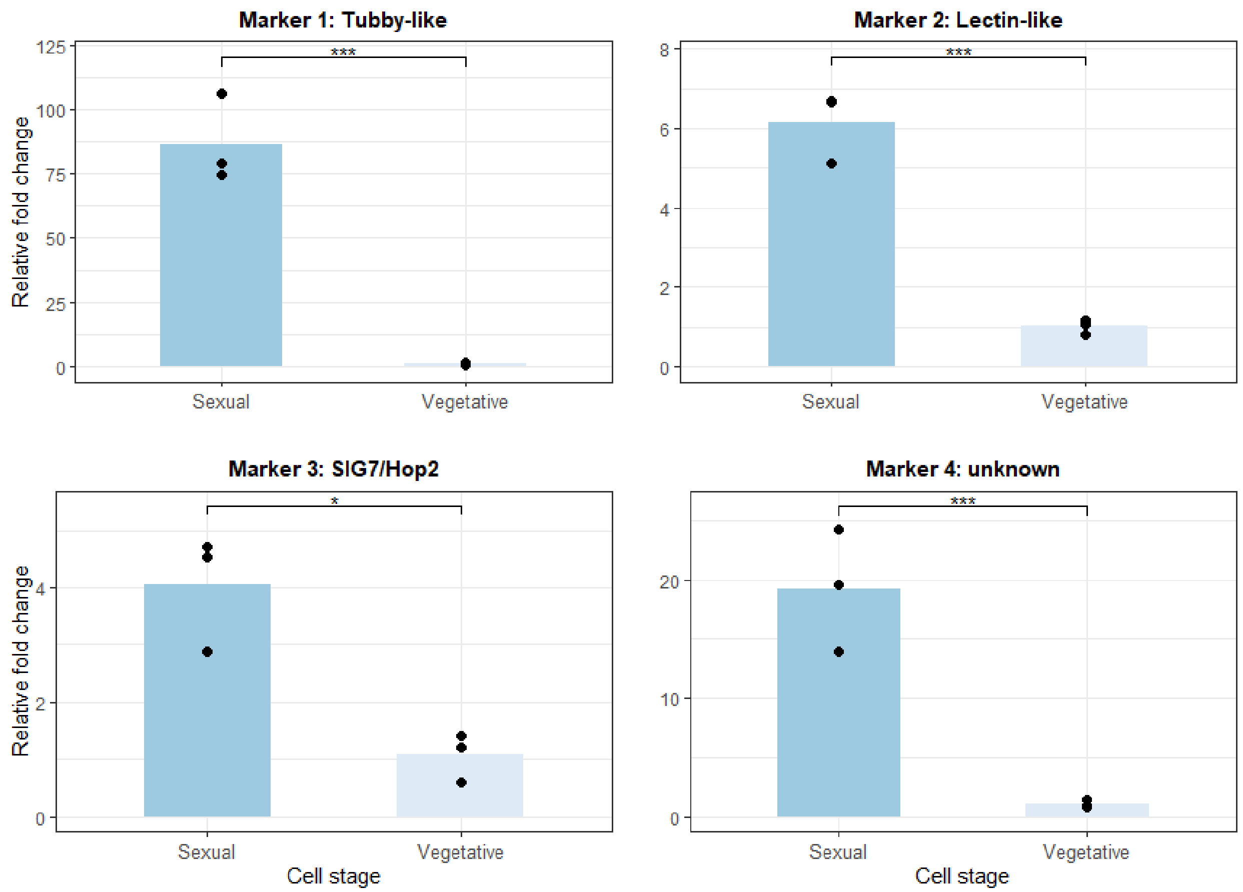

Supplementary Figure 6: Bar plots showing the relative fold changes obtained from qPCR data of four marker genes in sexual crosses and vegetative cultures of *Cylindrotheca closterium* strains A6 and MC4 (n = 3). Bars show the average relative expression, while dots show individual data points.

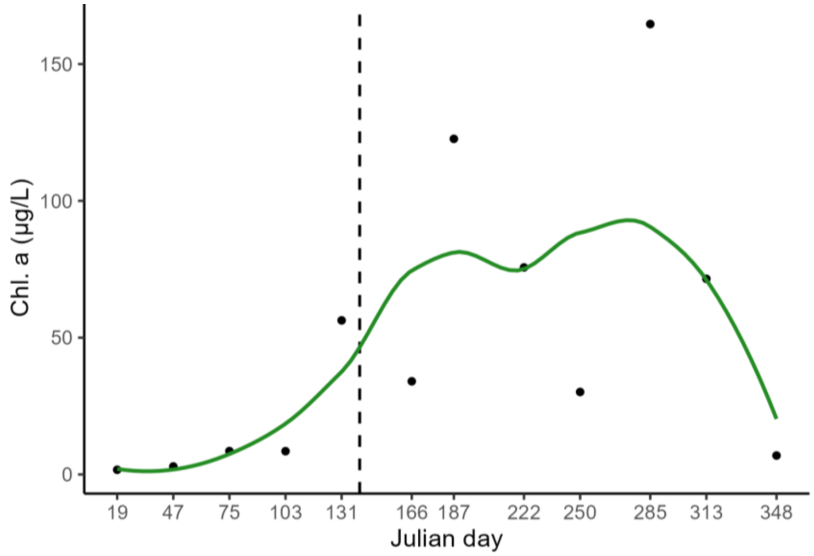

Supplementary Figure 7: Scatterplot of chlorophyll *a* concentrations during the 2022 bloom in the freshwater part of the Scheldt estuary near Melle. The green line shows chlorophyll trends as a Loess smoother. The sampling date for the metatranscriptome analysis is indicated with a dashed line.

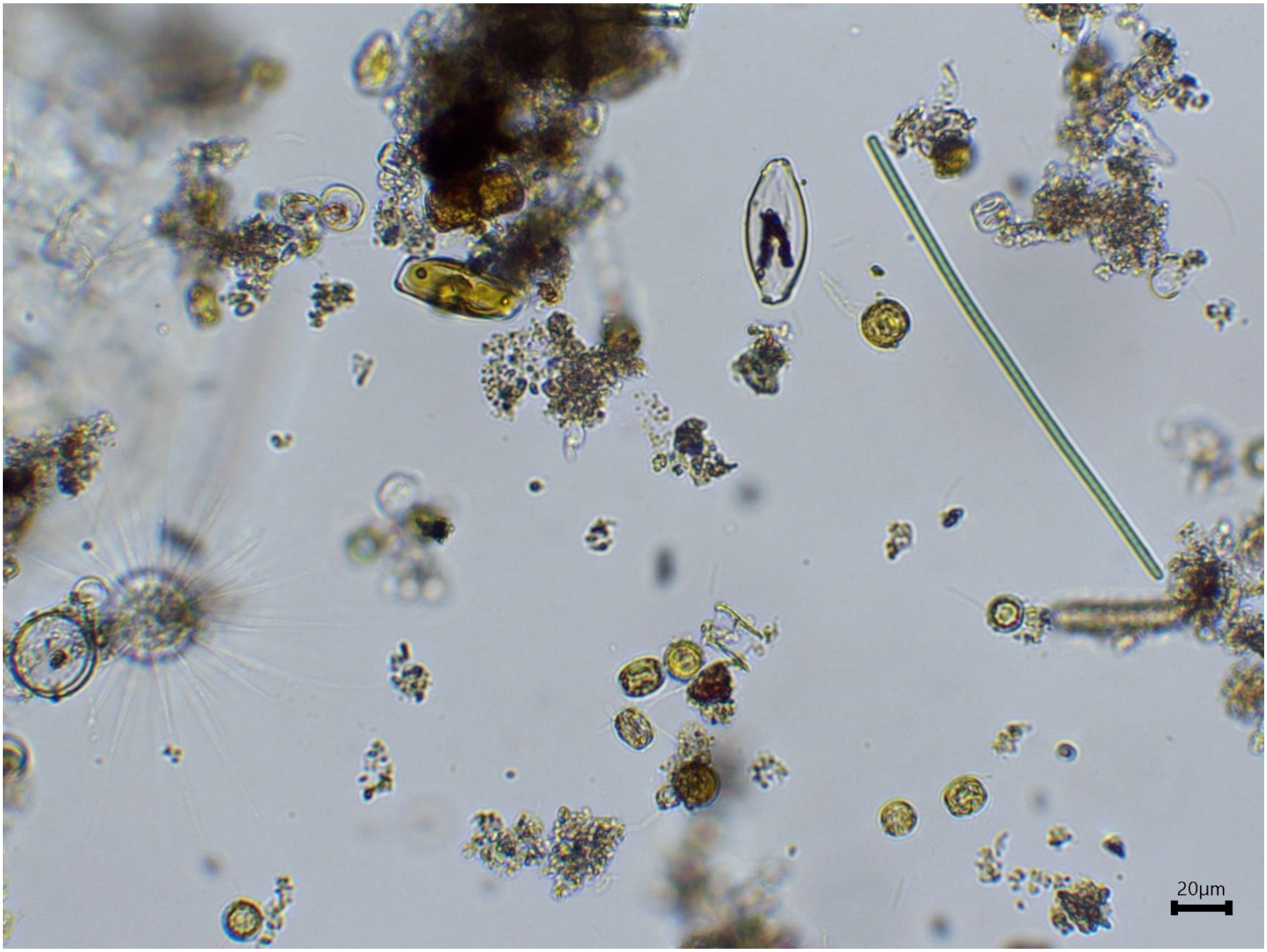

Supplementary Figure 8: Representative microscopic image of untreated microcosm community (control condition for metatranscriptome experiment). Scale bar: 20 µm.

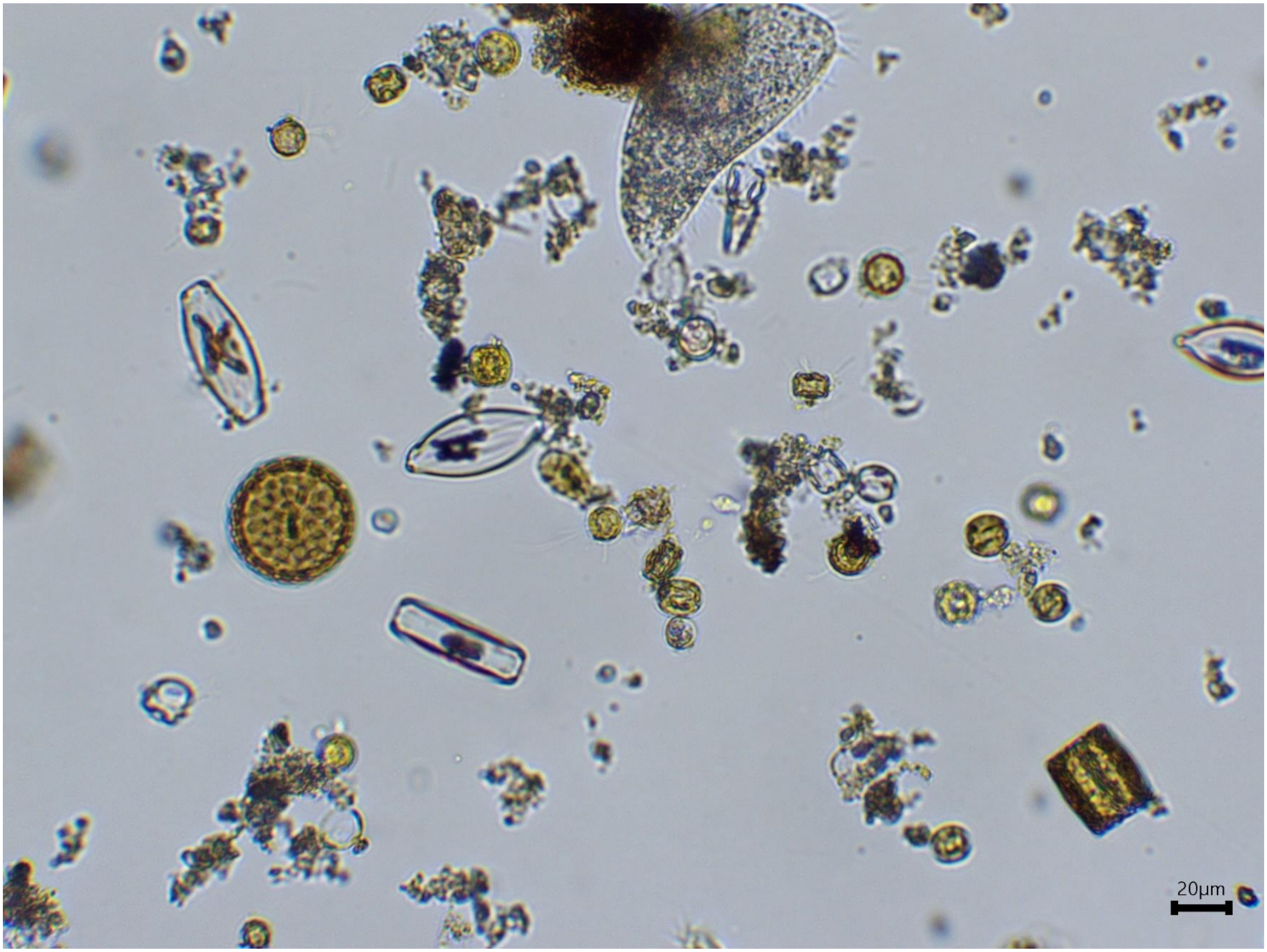

Supplementary Figure 9: Representative microscopic image of untreated microcosm community (control condition for metatranscriptome experiment). Scale bar: 20 µm.

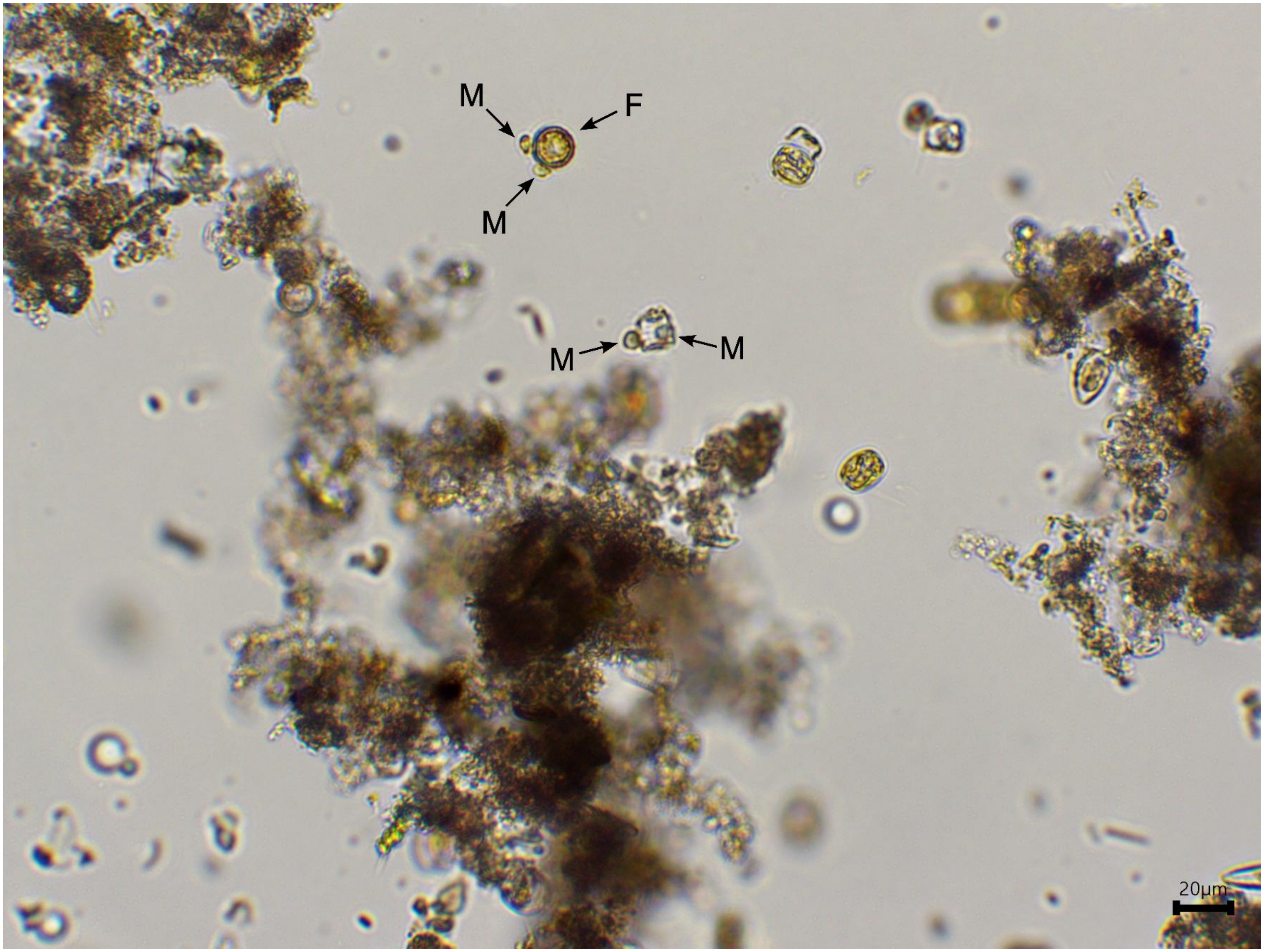

Supplementary Figure 10: Representative microscopic image of microcosm community after 24h of 10 ppt salt-treatment. Arrows indicate male spermatocytes and sperm cells (M) and female gametes (F). Scale bar: 20 µm.

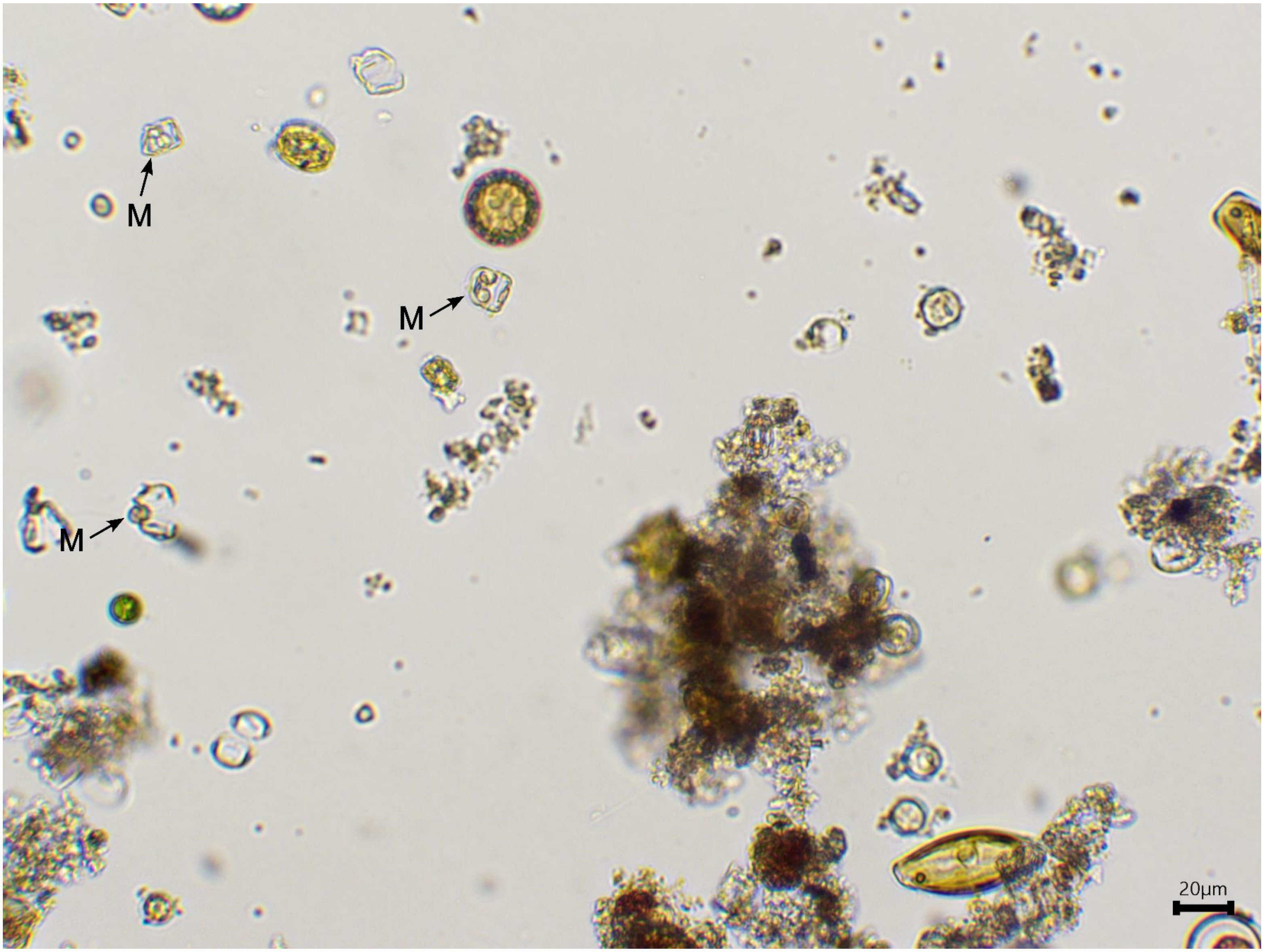

Supplementary Figure 11: Representative microscopic image of microcosm community after 24h of 10 ppt salt-treatment. Arrows indicate male spermatocytes and sperm cells (M). Scale bar: 20 µm.

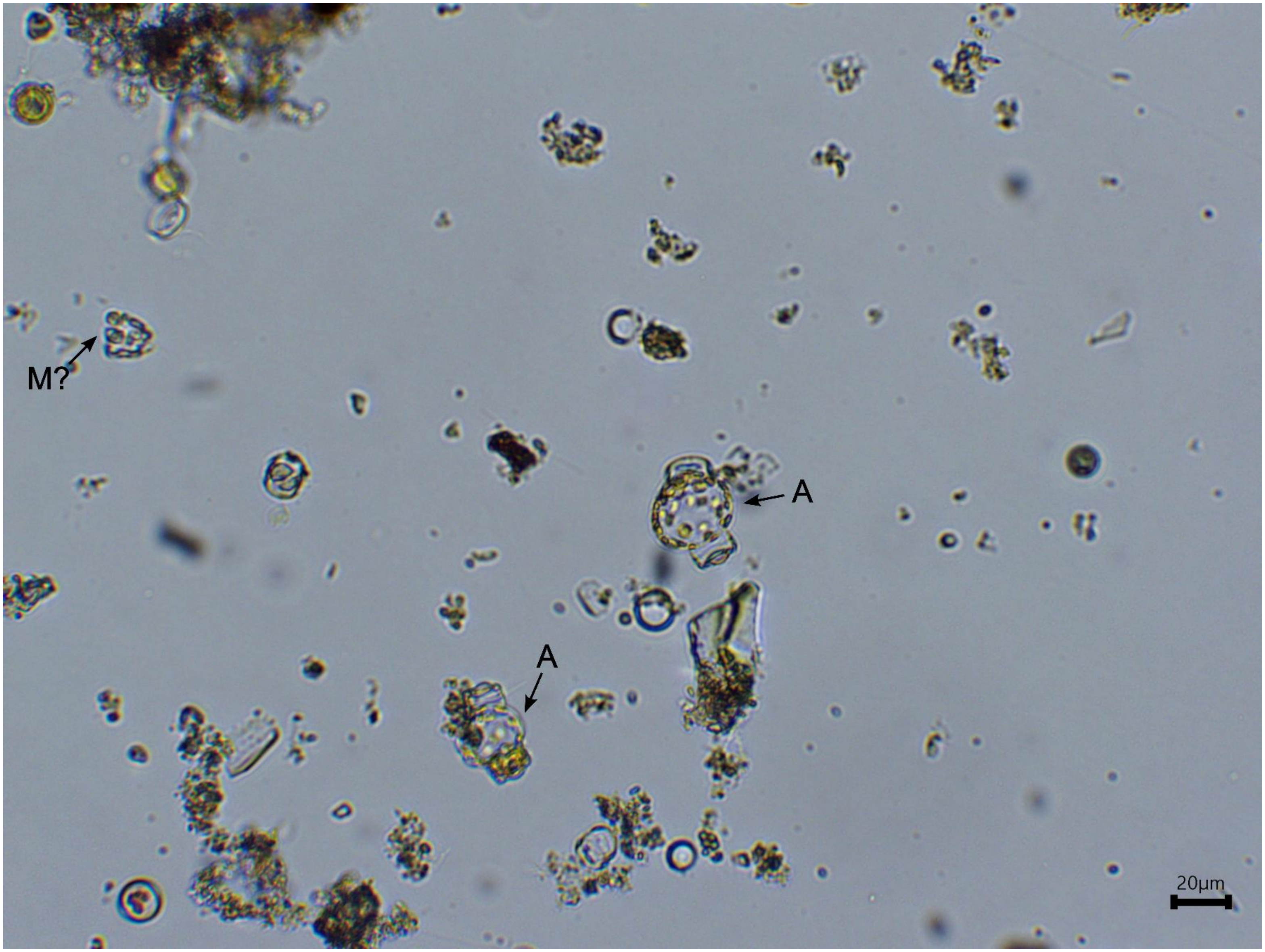

Supplementary Figure 12: Representative microscopic image of microcosm community after 48h of 10 ppt salt-treatment. Arrows indicate putative male sperm cells (M?) and auxospores (A). Scale bar: 20 µm.

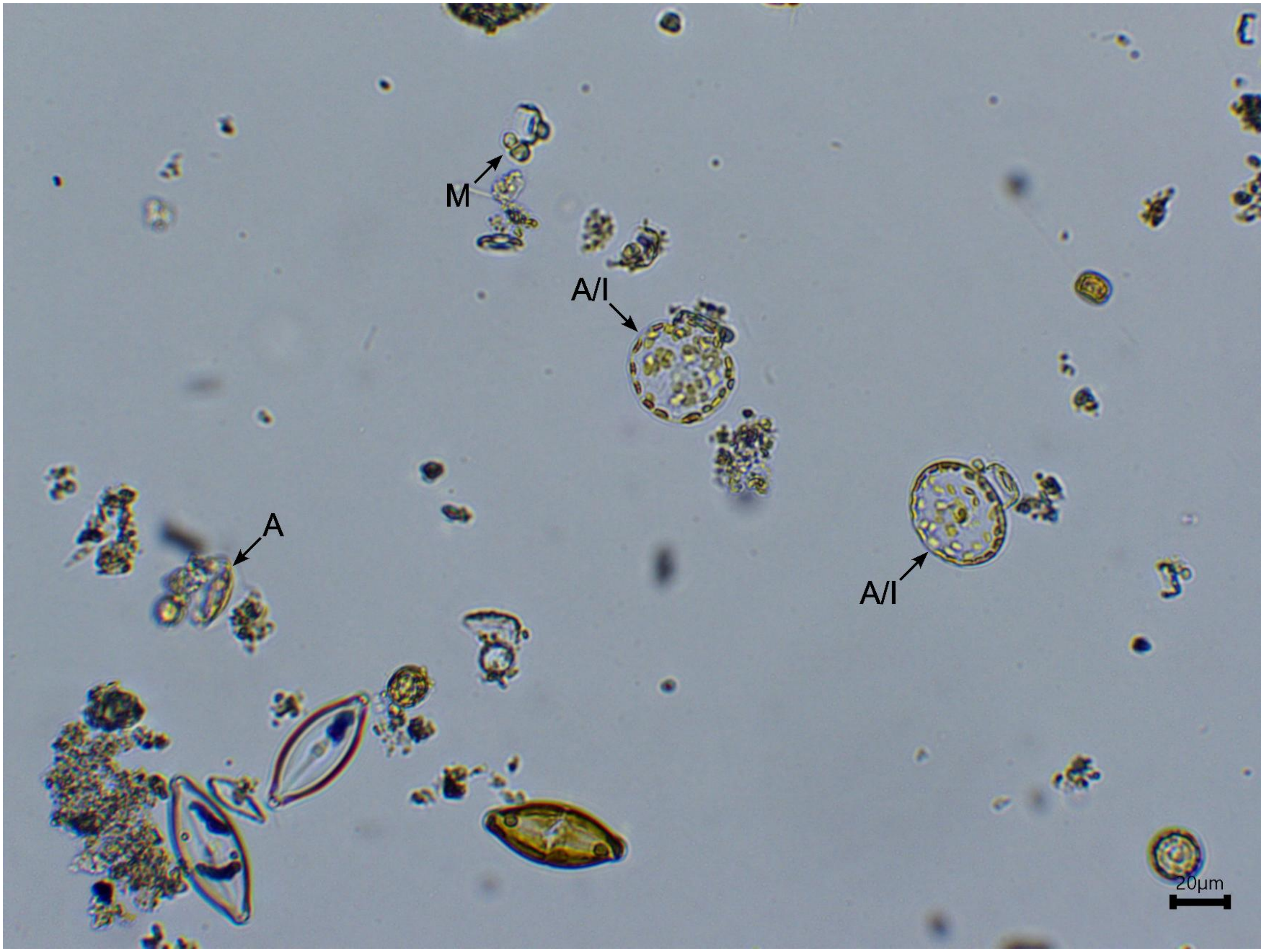

Supplementary Figure 13: Representative microscopic image of microcosm community after 48h of 10 ppt salt-treatment. Arrows indicate male sperm cells (M), auxospores (A) and mature auxospores or initial cells (A/I). Scale bar: 20 µm.

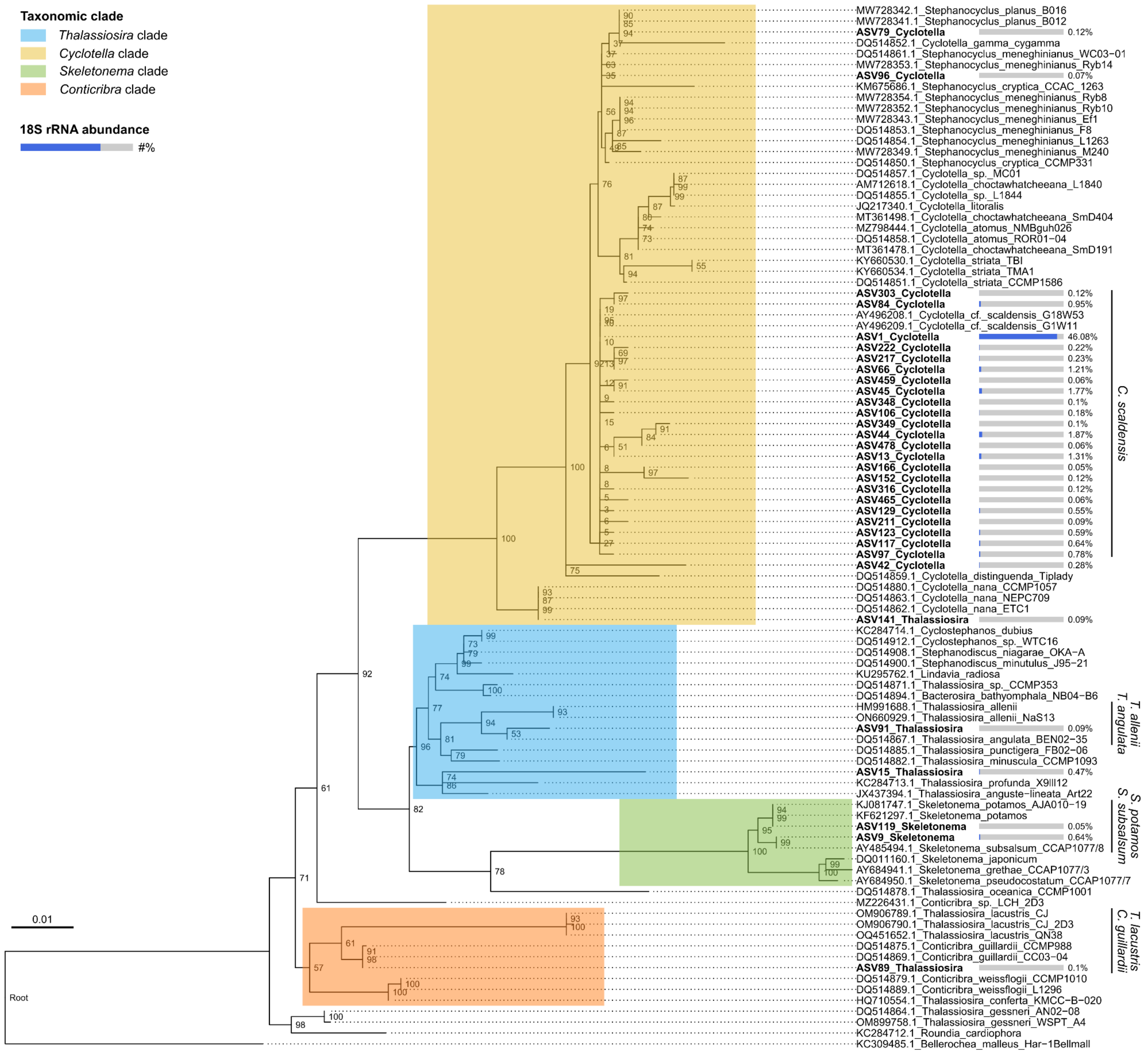

Taxonomic clade
Thalassiosira clade
Cyclotella clade
Skeletonema clade
Conticribra clade
18S rRNA abundance
#%
0.01
Root
MW728342.1_Stephanocyclus_planus_B016
MW728341.1_Stephanocyclus_planus_B012
ASV79_Cyclotella 0.12%
DQ514852.1_Cyclotella_gamma_cygamma
DQ514861.1_Stephanocyclus_meneghinianus_WC03−01
MW728353.1_Stephanocyclus_meneghinianus_Ryb14
ASV96_Cyclotella 0.07%
KM675686.1_Stephanocyclus_cryptica_CCAC_1263
MW728354.1_Stephanocyclus_meneghinianus_Ryb8
MW728352.1_Stephanocyclus_meneghinianus_Ryb10
MW728343.1_Stephanocyclus_meneghinianus_Ef1
DQ514853.1_Stephanocyclus_meneghinianus_F8
DQ514854.1_Stephanocyclus_meneghinianus_L1263
MW728349.1_Stephanocyclus_meneghinianus_M240
DQ514850.1_Stephanocyclus_cryptica_CCMP331
DQ514857.1_Cyclotella_sp._MC01
AM712618.1_Cyclotella_choctawhatcheeana_L1840
DQ514855.1_Cyclotella_sp._L1844
JQ217340.1_Cyclotella_litoralis
MT361498.1_Cyclotella_choctawhatcheeana_SmD404
MZ798444.1_Cyclotella_atomus_NMBguh026
DQ514858.1_Cyclotella_atomus_ROR01−04
MT361478.1_Cyclotella_choctawhatcheeana_SmD191
KY660530.1_Cyclotella_striata_TBI
KY660534.1_Cyclotella_striata_TMA1
DQ514851.1_Cyclotella_striata_CCMP1586
ASV303_Cyclotella 0.12%
ASV84_Cyclotella 0.95%
AY496208.1_Cyclotella_cf._scaldensis_G18W53
AY496209.1_Cyclotella_cf._scaldensis_G1W11
ASV1_Cyclotella 46.08%
ASV222_Cyclotella 0.22%
ASV217_Cyclotella 0.23%
ASV66_Cyclotella 1.21%
ASV459_Cyclotella 0.06%
ASV45_Cyclotella 1.77%
ASV348_Cyclotella 0.1%
ASV106_Cyclotella 0.18%
ASV349_Cyclotella 0.1%
ASV44_Cyclotella 1.87%
ASV478_Cyclotella 0.06%
ASV13_Cyclotella 1.31%
ASV166_Cyclotella 0.05%
ASV152_Cyclotella 0.12%
ASV316_Cyclotella 0.12%
ASV465_Cyclotella 0.06%
ASV129_Cyclotella 0.55%
ASV211_Cyclotella 0.09%
ASV123_Cyclotella 0.59%
ASV117_Cyclotella 0.64%
ASV97_Cyclotella 0.78%
ASV42_Cyclotella 0.28%
C. scaldensis
DQ514859.1_Cyclotella_distinguenda_Tiplady
DQ514880.1_Cyclotella_nana_CCMP1057
DQ514863.1_Cyclotella_nana_NEPC709
DQ514862.1_Cyclotella_nana_ETC1
ASV141_Thalassiosira 0.09%
KC284714.1_Cyclostephanos_dubius
DQ514912.1_Cyclostephanos_sp._WTC16
DQ514908.1_Stephanodiscus_niagarae_OKA−A
DQ514900.1_Stephanodiscus_minutulus_J95−21
KU295762.1_Lindavia_radiosa
DQ514871.1_Thalassiosira_sp._CCMP353
DQ514894.1_Bacterosira_bathyomphala_NB04−B6
HM991688.1_Thalassiosira_allenii
ON660929.1_Thalassiosira_allenii_NaS13
ASV91_Thalassiosira 0.09%
DQ514867.1_Thalassiosira_angulata_BEN02−35
DQ514885.1_Thalassiosira_punctigera_FB02−06
DQ514882.1_Thalassiosira_minuscula_CCMP1093
ASV15_Thalassiosira 0.47%
KC284713.1_Thalassiosira_profunda_X9lll12
JX437394.1_Thalassiosira_anguste−lineata_Art22
T. allenii
T. angulata
KJ081747.1_Skeletonema_potamos_AJA010−19
KF621297.1_Skeletonema_potamos
ASV119_Skeletonema 0.05%
ASV9_Skeletonema 0.64%
AY485494.1_Skeletonema_subsalsum_CCAP1077/8
DQ011160.1_Skeletonema_japonicum
AY684941.1_Skeletonema_grethae_CCAP1077/3
AY684950.1_Skeletonema_pseudocostatum_CCAP1077/7
S. potamos
S. subsalsum
DQ514878.1_Thalassiosira_oceanica_CCMP1001
MZ226431.1_Conticribra_sp._LCH_2D3
OM906789.1_Thalassiosira_lacustris_CJ
OM906790.1_Thalassiosira_lacustris_CJ_2D3
OQ451652.1_Thalassiosira_lacustris_QN38
DQ514875.1_Conticribra_guillardii_CCMP988
DQ514869.1_Conticribra_guillardii_CC03−04
ASV89_Thalassiosira 0.1%
T. lacustris
C. guillardii
DQ514879.1_Conticribra_weissflogii_CCMP1010
DQ514889.1_Conticribra_weissflogii_L1296
HQ710554.1_Thalassiosira_conferta_KMCC−B−020
DQ514864.1_Thalassiosira_gessneri_AN02−08
OM899758.1_Thalassiosira_gessneri_WSPT_A4
KC284712.1_Roundia_cardiophora
KC309485.1_Bellerochea_malleus_Har−1Bellmall

Supplementary Figure 14: Bootstrap-consensus phylogenetic tree of diatom 18S ASV sequences observed during the microcosm experiment as well as reference Thalassiosirales sequences. For each reference sequence, the NCBI GenBank identifier is displayed in front of the species name. Bar plots next to the ASV names show the average abundance of each ASV in the dataset.

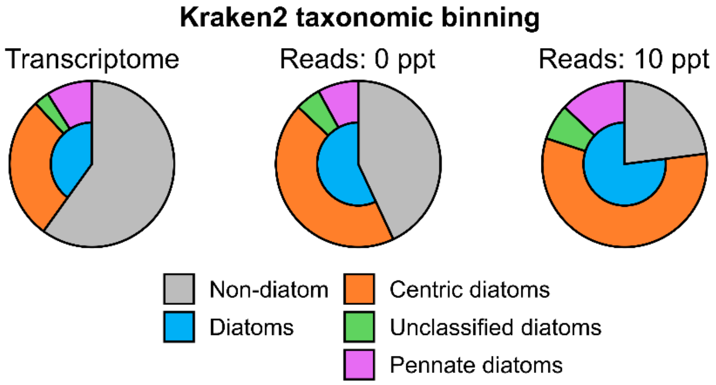

Supplementary Figure 15: Pie charts showing the proportion of transcripts and raw Illumina reads that could be taxonomically annotated as belonging to pennate and centric diatoms. Transcripts and reads that could not be classified to any taxon level were omitted.

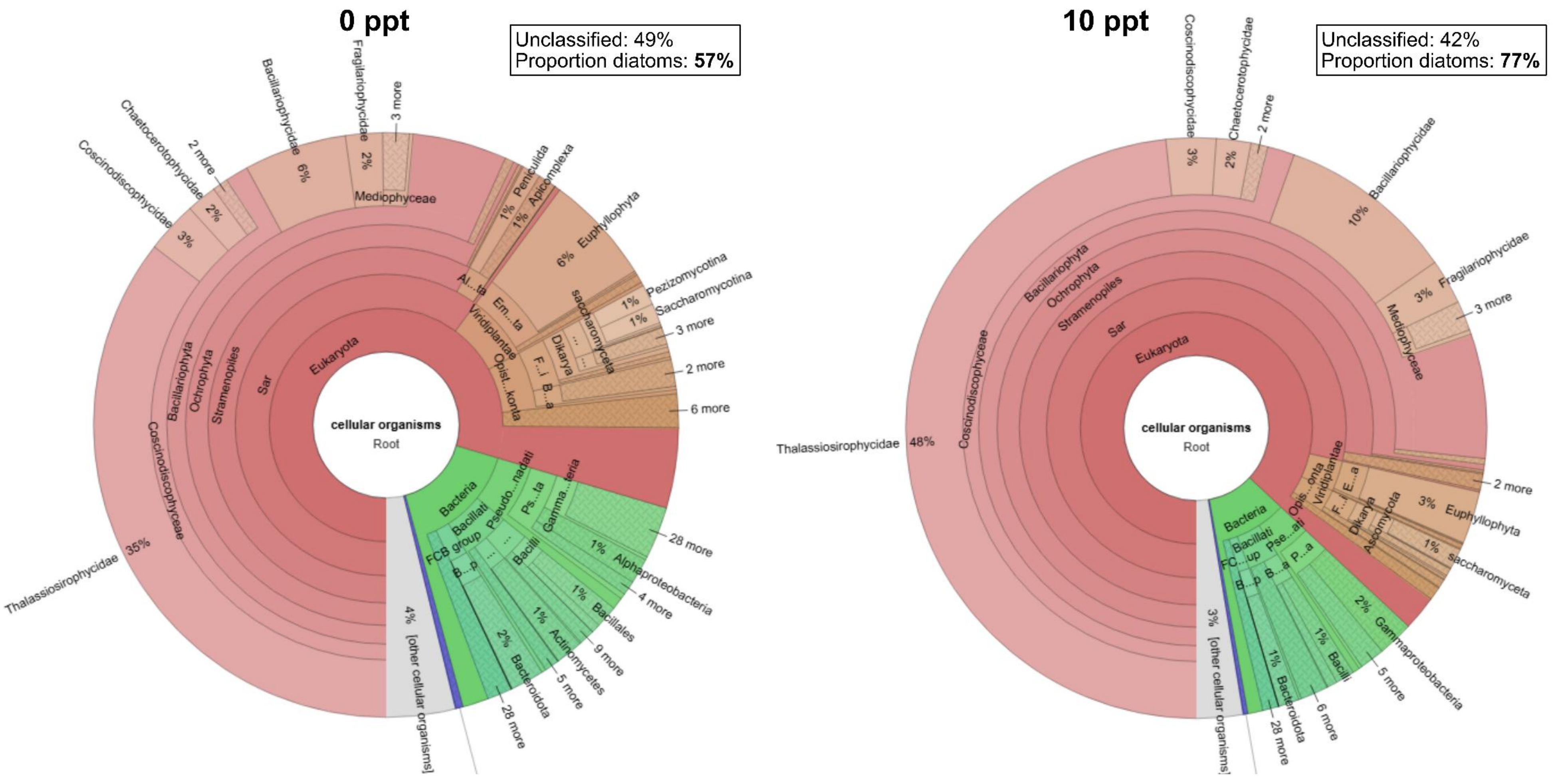

Supplementary Figure 16: Krona-plots showing the taxonomic distribution of RNA-seq reads in control (0 ppt) and salinity-treated (10 ppt) cultures as determined by Kraken2. The reported proportion of diatoms is relative to all cellular organisms reads.

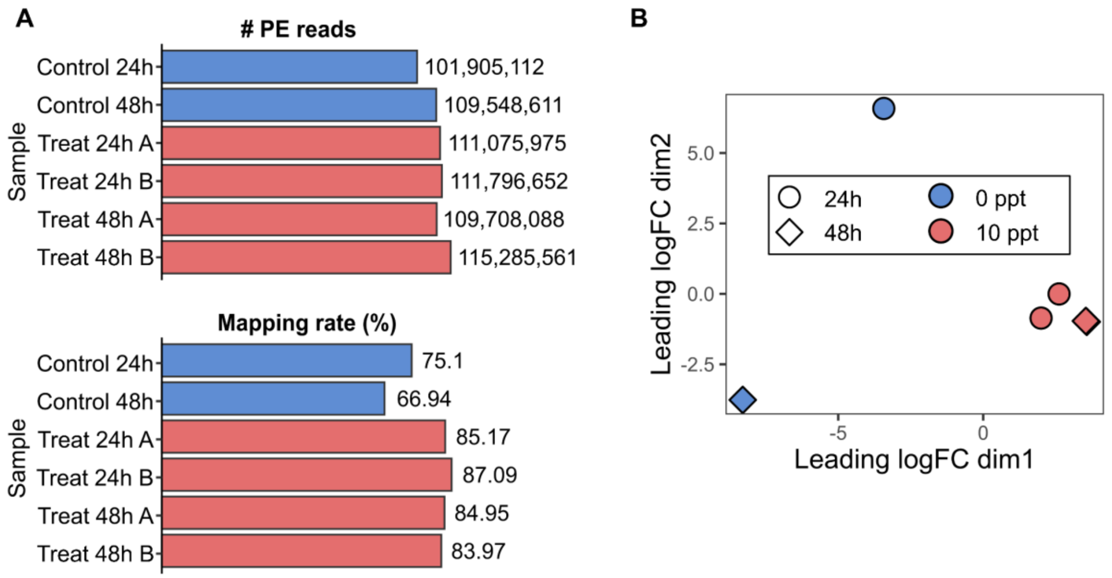

Supplementary Figure 17: RNA-sequencing of microcosm experiment. (A) Library size and mapping rates of six metatranscriptome libraries. PE: paired-end. **(B)** Multidimensional scaling plot comparing expression between six metatranscriptomic samples covering two time points and salinities.

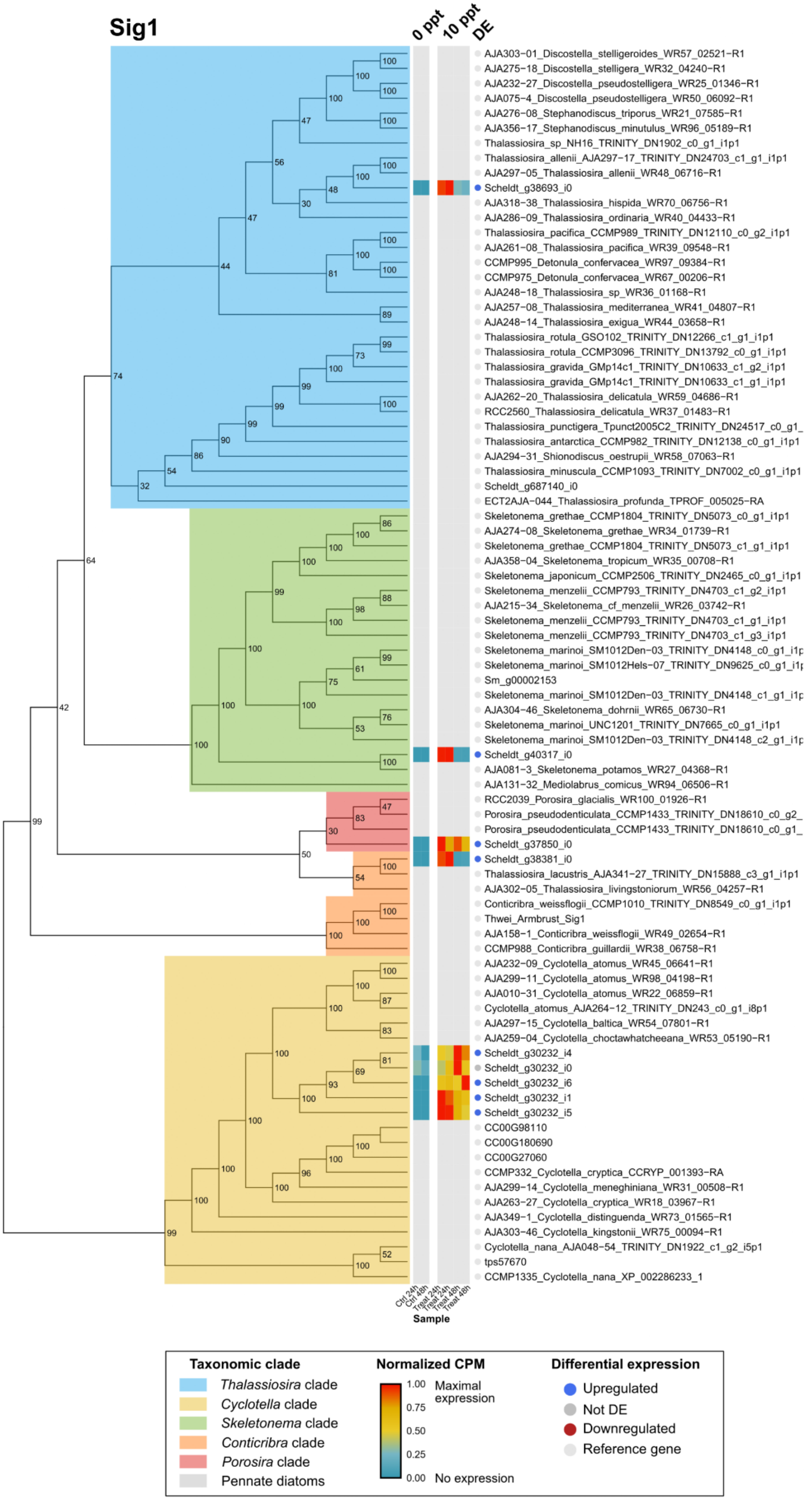
Sig1
0 ppt
10 ppt
DE
AJA303-01_Discostella_stelligeroides_WR57_02521-R1
AJA275-18_Discostella_stelligera_WR32_04240-R1
AJA232-27_Discostella_pseudostelligera_WR25_01346-R1
AJA075-4_Discostella_pseudostelligera_WR50_06092-R1
AJA276-08_Stephanodiscus_triporus_WR21_07585-R1
AJA356-17_Stephanodiscus_minutulus_WR96_05189-R1
Thalassiosira_sp_NH16_TRINITY_DN1902_c0_g1_i1p1
Thalassiosira_allenii_AJA297-17_TRINITY_DN24703_c1_g1_i1p1
AJA297-05_Thalassiosira_allenii_WR48_06716-R1
Scheldt_g38693_i0
AJA318-38_Thalassiosira_hispida_WR70_06756-R1
AJA286-09_Thalassiosira_ordinaria_WR40_04433-R1
Thalassiosira_pacifica_CCMP989_TRINITY_DN12110_c0_g2_i1p1
AJA261-08_Thalassiosira_pacifica_WR39_09548-R1
CCMP995_Detonula_confervacea_WR97_09384-R1
CCMP975_Detonula_confervacea_WR67_00206-R1
AJA248-18_Thalassiosira_sp_WR36_01168-R1
AJA257-08_Thalassiosira_mediterranea_WR41_04807-R1
AJA248-14_Thalassiosira_exigua_WR44_03658-R1
Thalassiosira_rotula_GSO102_TRINITY_DN12266_c1_g1_i1p1
Thalassiosira_rotula_CCMP3096_TRINITY_DN13792_c0_g1_i1p1
Thalassiosira_gravida_GMp14c1_TRINITY_DN10633_c1_g2_i1p1
Thalassiosira_gravida_GMp14c1_TRINITY_DN10633_c1_g1_i1p1
AJA262-20_Thalassiosira_delicatula_WR59_04686-R1
RCC2560_Thalassiosira_delicatula_WR37_01483-R1
Thalassiosira_punctigera_Tpunct2005C2_TRINITY_DN24517_c0_g1_
Thalassiosira_antarctica_CCMP982_TRINITY_DN12138_c0_g1_i1p1
AJA294-31_Shionodiscus_oestrupii_WR58_07063-R1
Thalassiosira_minuscula_CCMP1093_TRINITY_DN7002_c0_g1_i1p1
Scheldt_g687140_i0
ECT2AJA-044_Thalassiosira_profunda_TPROF_005025-RA
Skeletonema_grethae_CCMP1804_TRINITY_DN5073_c0_g1_i1p1
AJA274-08_Skeletonema_grethae_WR34_01739-R1
Skeletonema_grethae_CCMP1804_TRINITY_DN5073_c1_g1_i1p1
AJA358-04_Skeletonema_tropicum_WR35_00708-R1
Skeletonema_japonicum_CCMP2506_TRINITY_DN2465_c0_g1_i1p1
Skeletonema_menzelii_CCMP793_TRINITY_DN4703_c1_g2_i1p1
AJA215-34_Skeletonema_cf_menzelii_WR26_03742-R1
Skeletonema_menzelii_CCMP793_TRINITY_DN4703_c1_g1_i1p1
Skeletonema_menzelii_CCMP793_TRINITY_DN4703_c1_g3_i1p1
Skeletonema_marinoi_SM1012Den-03_TRINITY_DN4148_c0_g1_i1p
Skeletonema_marinoi_SM1012Hels-07_TRINITY_DN9625_c0_g1_i1p
Sm_g00002153
Skeletonema_marinoi_SM1012Den-03_TRINITY_DN4148_c1_g1_i1p
AJA304-46_Skeletonema_dohrnii_WR65_06730-R1
Skeletonema_marinoi_UNC1201_TRINITY_DN7665_c0_g1_i1p1
Skeletonema_marinoi_SM1012Den-03_TRINITY_DN4148_c2_g1_i1p
Scheldt_g40317_i0
AJA081-3_Skeletonema_potamos_WR27_04368-R1
AJA131-32_Mediolabrus_comicus_WR94_06506-R1
RCC2039_Porosira_glacialis_WR100_01926-R1
Porosira_pseudodenticulata_CCMP1433_TRINITY_DN18610_c0_g2_
Porosira_pseudodenticulata_CCMP1433_TRINITY_DN18610_c0_g1_
Scheldt_g37850_i0
Scheldt_g38381_i0
Thalassiosira_lacustris_AJA341-27_TRINITY_DN15888_c3_g1_i1p1
AJA302-05_Thalassiosira_livingstoniorum_WR56_04257-R1
Conticribra_weissflogii_CCMP1010_TRINITY_DN8549_c0_g1_i1p1
Thwei_Armbrust_Sig1
AJA158-1_Conticribra_weissflogii_WR49_02654-R1
CCMP988_Conticribra_guillardii_WR38_06758-R1
AJA232-09_Cyclotella_atomus_WR45_06641-R1
AJA299-11_Cyclotella_atomus_WR98_04198-R1
AJA010-31_Cyclotella_atomus_WR22_06859-R1
Cyclotella_atomus_AJA264-12_TRINITY_DN243_c0_g1_i8p1
AJA297-15_Cyclotella_baltica_WR54_07801-R1
AJA259-04_Cyclotella_choctawhatcheeana_WR53_05190-R1
Scheldt_g30232_i4
Scheldt_g30232_i0
Scheldt_g30232_i6
Scheldt_g30232_i1
Scheldt_g30232_i5
CC00G98110
CC00G180690
CC00G27060
CCMP332_Cyclotella_cryptica_CCRYP_001393-RA
AJA299-14_Cyclotella_meneghiniana_WR31_00508-R1
AJA263-27_Cyclotella_cryptica_WR18_03967-R1
AJA349-1_Cyclotella_distinguenda_WR73_01565-R1
AJA303-46_Cyclotella_kingstonii_WR75_00094-R1
Cyclotella_nana_AJA048-54_TRINITY_DN1922_c1_g2_i5p1
tps57670
CCMP1335_Cyclotella_nana_XP_002286233_1
Ctrl 24h
Ctrl 48h
Treat 24h
Treat 48h
Treat 24h
Treat 48h
Sample
Taxonomic clade
Thalassiosira clade
Cyclotella clade
Skeletonema clade
Conticribra clade
Porosira clade
Pennate diatoms
Normalized CPM
1.00
0.75
0.50
0.25
0.00
Maximal expression
No expression
Differential expression
Upregulated
Not DE
Downregulated
Reference gene

Supplementary Figure 18: Cladogram of midpoint-rooted bootstrap consensus phylogenetic tree of Sig1 protein sequences from the Scheldt microcosm metatranscriptome as well as reference Sig1 proteins from Roberts et al. (2023), Pinder et al., in prep., Audoor et al. (2024) and the PLAZA Diatoms v1.0 platform (Osuna-Cruz et al. 2020). Clades within the Thalassiosirales follow Roberts et al. (2023). Next to the phylogeny, a heatmap shows the expression of Scheldt transcripts as normalized counts per million (CPM) in control (0 ppt) and salt-treated (10 ppt) conditions. Ppt = parts per thousand.

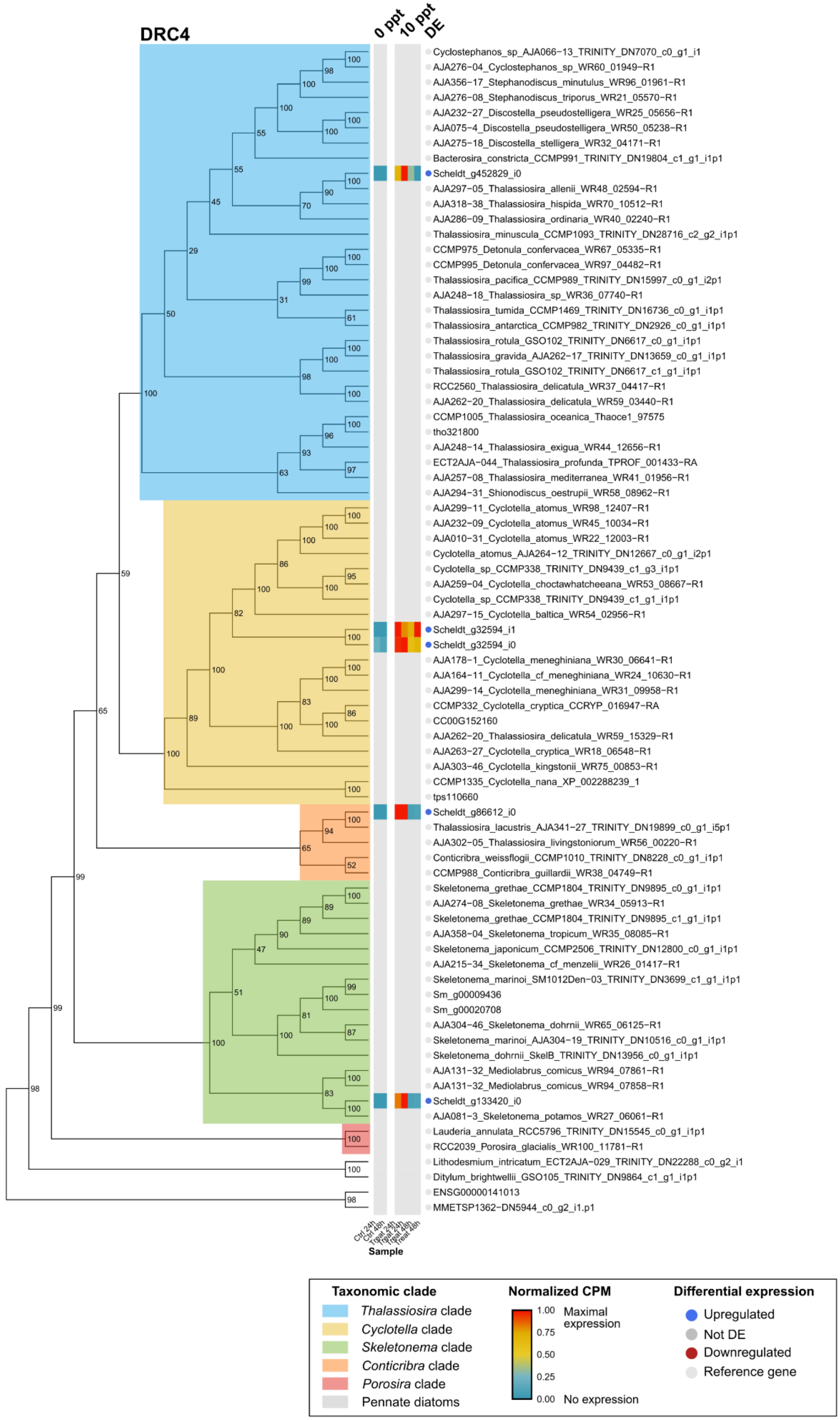
DRC4
0 ppt
10 ppt
DE
Cyclostephanos_sp_AJA066−13_TRINITY_DN7070_c0_g1_i1
AJA276−04_Cyclostephanos_sp_WR60_01949−R1
AJA356−17_Stephanodiscus_minutulus_WR96_01961−R1
AJA276−08_Stephanodiscus_triporus_WR21_05570−R1
AJA232−27_Discostella_pseudostelligera_WR25_05656−R1
AJA075−4_Discostella_pseudostelligera_WR50_05238−R1
AJA275−18_Discostella_stelligera_WR32_04171−R1
Bacterosira_constricta_CCMP991_TRINITY_DN19804_c1_g1_i1p1
Scheldt_g452829_i0
AJA297−05_Thalassiosira_allenii_WR48_02594−R1
AJA318−38_Thalassiosira_hispida_WR70_10512−R1
AJA286−09_Thalassiosira_ordinaria_WR40_02240−R1
Thalassiosira_minuscula_CCMP1093_TRINITY_DN28716_c2_g2_i1p1
CCMP975_Detonula_confervacea_WR67_05335−R1
CCMP995_Detonula_confervacea_WR97_04482−R1
Thalassiosira_pacifica_CCMP989_TRINITY_DN15997_c0_g1_i2p1
AJA248−18_Thalassiosira_sp_WR36_07740−R1
Thalassiosira_tumida_CCMP1469_TRINITY_DN16736_c0_g1_i1p1
Thalassiosira_antarctica_CCMP982_TRINITY_DN2926_c0_g1_i1p1
Thalassiosira_rotula_GSO102_TRINITY_DN6617_c0_g1_i1p1
Thalassiosira_gravida_AJA262−17_TRINITY_DN13659_c0_g1_i1p1
Thalassiosira_rotula_GSO102_TRINITY_DN6617_c1_g1_i1p1
RCC2560_Thalassiosira_delicatula_WR37_04417−R1
AJA262−20_Thalassiosira_delicatula_WR59_03440−R1
CCMP1005_Thalassiosira_oceanica_Thaoce1_97575
tho321800
AJA248−14_Thalassiosira_exigua_WR44_12656−R1
ECT2AJA−044_Thalassiosira_profunda_TPROF_001433−RA
AJA257−08_Thalassiosira_mediterranea_WR41_01956−R1
AJA294−31_Shionodiscus_oestrupii_WR58_08962−R1
AJA299−11_Cyclotella_atomus_WR98_12407−R1
AJA232−09_Cyclotella_atomus_WR45_10034−R1
AJA010−31_Cyclotella_atomus_WR22_12003−R1
Cyclotella_atomus_AJA264−12_TRINITY_DN12667_c0_g1_i2p1
Cyclotella_sp_CCMP338_TRINITY_DN9439_c1_g3_i1p1
AJA259−04_Cyclotella_choctawhatcheeana_WR53_08667−R1
Cyclotella_sp_CCMP338_TRINITY_DN9439_c1_g1_i1p1
AJA297−15_Cyclotella_baltica_WR54_02956−R1
Scheldt_g32594_i1
Scheldt_g32594_i0
AJA178−1_Cyclotella_meneghiniana_WR30_06641−R1
AJA164−11_Cyclotella_cf_meneghiniana_WR24_10630−R1
AJA299−14_Cyclotella_meneghiniana_WR31_09958−R1
CCMP332_Cyclotella_cryptica_CCRYP_016947−RA
CC00G152160
AJA262−20_Thalassiosira_delicatula_WR59_15329−R1
AJA263−27_Cyclotella_cryptica_WR18_06548−R1
AJA303−46_Cyclotella_kingstonii_WR75_00853−R1
CCMP1335_Cyclotella_nana_XP_002288239_1
tps110660
Scheldt_g86612_i0
Thalassiosira_lacustris_AJA341−27_TRINITY_DN19899_c0_g1_i5p1
AJA302−05_Thalassiosira_livingstoniorum_WR56_00220−R1
Conticribra_weissflogii_CCMP1010_TRINITY_DN8228_c0_g1_i1p1
CCMP988_Conticribra_guillardii_WR38_04749−R1
Skeletonema_grethae_CCMP1804_TRINITY_DN9895_c0_g1_i1p1
AJA274−08_Skeletonema_grethae_WR34_05913−R1
Skeletonema_grethae_CCMP1804_TRINITY_DN9895_c1_g1_i1p1
AJA358−04_Skeletonema_tropicum_WR35_08085−R1
Skeletonema_japonicum_CCMP2506_TRINITY_DN12800_c0_g1_i1p1
AJA215−34_Skeletonema_cf_menzelii_WR26_01417−R1
Skeletonema_marinoi_SM1012Den−03_TRINITY_DN3699_c1_g1_i1p1
Sm_g00009436
Sm_g00020708
AJA304−46_Skeletonema_dohrnii_WR65_06125−R1
Skeletonema_marinoi_AJA304−19_TRINITY_DN10516_c0_g1_i1p1
Skeletonema_dohrnii_SkelB_TRINITY_DN13956_c0_g1_i1p1
AJA131−32_Mediolabrus_comicus_WR94_07861−R1
AJA131−32_Mediolabrus_comicus_WR94_07858−R1
Scheldt_g133420_i0
AJA081−3_Skeletonema_potamos_WR27_06061−R1
Lauderia_annulata_RCC5796_TRINITY_DN15545_c0_g1_i1p1
RCC2039_Porosira_glacialis_WR100_11781−R1
Lithodesmium_intricatum_ECT2AJA−029_TRINITY_DN22288_c0_g2_i1
Ditylum_brightwellii_GSO105_TRINITY_DN9864_c1_g1_i1p1
ENSG00000141013
MMETSP1362−DN5944_c0_g2_i1.p1
Ctrl 24h
Ctrl 48h
Treat 24h
Treat 48h
Ctrl 24h
Ctrl 48h
Treat 24h
Treat 48h
Sample
Taxonomic clade
Thalassiosira clade
Cyclotella clade
Skeletonema clade
Conticribra clade
Porosira clade
Pennate diatoms
Normalized CPM
1.00
0.75
0.50
0.25
0.00
Maximal expression
No expression
Differential expression
Upregulated
Not DE
Downregulated
Reference gene

Supplementary Figure 19: Cladogram of midpoint-rooted bootstrap consensus phylogenetic tree of DRC4 protein sequences from the Scheldt microcosm metatranscriptome as well as reference DRC4 proteins from Roberts et al. (2023), Pinder et al., in prep., Audoor et al. (2024) and the PLAZA Diatoms v1.0 platform (Osuna-Cruz et al. 2020). Clades within the Thalassiosirales follow Roberts et al. (2023). Next to the phylogeny, a heatmap shows the expression of Scheldt transcripts as normalized counts per million (CPM) in control (0 ppt) and salt-treated (10 ppt) conditions. Ppt = parts per thousand.

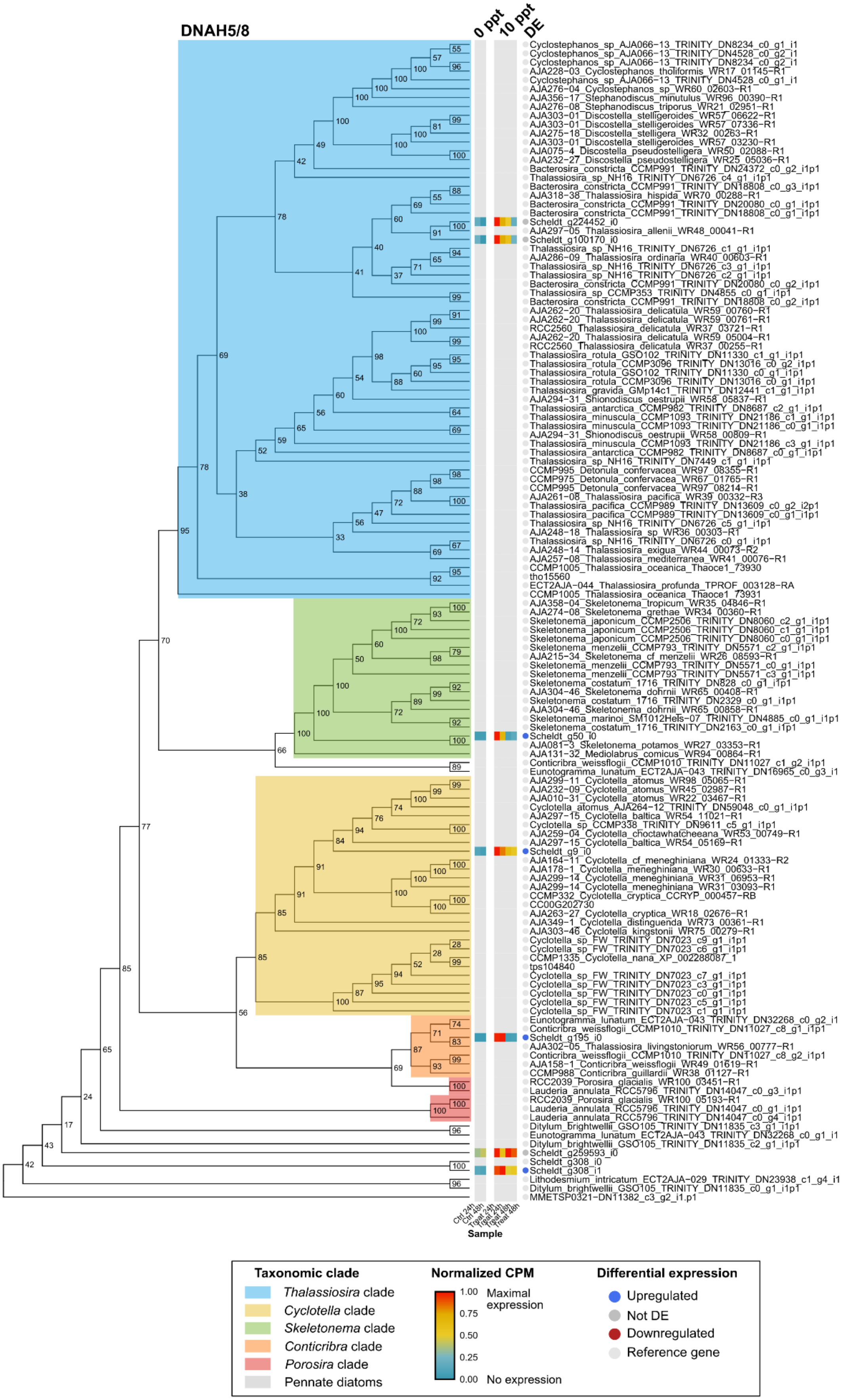
DNAH5/8
0 ppt
10 ppt
DE
Cyclostephanos_sp_AJA066-13_TRINITY_DN8234_c0_g1_i1
Cyclostephanos_sp_AJA066-13_TRINITY_DN4528_c0_g2_i1
Cyclostephanos_sp_AJA066-13_TRINITY_DN8234_c0_g2_i1
AJA228-03_Cyclostephanos_tholiformis_WR17_01145-R1
Cyclostephanos_sp_AJA066-13_TRINITY_DN4528_c0_g1_i1
AJA276-04_Cyclostephanos_sp_WR60_02603-R1
AJA356-17_Stephanodiscus_minutulus_WR96_00390-R1
AJA276-08_Stephanodiscus_triporus_WR21_02951-R1
AJA303-01_Discostella_stelligeroides_WR57_06622-R1
AJA303-01_Discostella_stelligeroides_WR57_07336-R1
AJA275-18_Discostella_stelligera_WR32_00263-R1
AJA303-01_Discostella_stelligeroides_WR57_03230-R1
AJA075-4_Discostella_pseudostelligera_WR50_02088-R1
AJA232-27_Discostella_pseudostelligera_WR25_05036-R1
Bacterosira_constricta_CCMP991_TRINITY_DN24372_c0_g2_i1p1
Thalassiosira_sp_NH16_TRINITY_DN6726_c4_g1_i1p1
Bacterosira_constricta_CCMP991_TRINITY_DN18808_c0_g3_i1p1
AJA318-38_Thalassiosira_hispida_WR70_00288-R1
Bacterosira_constricta_CCMP991_TRINITY_DN20080_c0_g1_i1p1
Bacterosira_constricta_CCMP991_TRINITY_DN18808_c0_g1_i1p1
Scheldt_g224452_i0
AJA297-05_Thalassiosira_allenii_WR48_00041-R1
Scheldt_g100170_i0
Thalassiosira_sp_NH16_TRINITY_DN6726_c1_g1_i1p1
AJA286-09_Thalassiosira_ordinaria_WR40_00603-R1
Thalassiosira_sp_NH16_TRINITY_DN6726_c3_g1_i1p1
Thalassiosira_sp_NH16_TRINITY_DN6726_c2_g1_i1p1
Bacterosira_constricta_CCMP991_TRINITY_DN20080_c0_g2_i1p1
Thalassiosira_sp_CCMP353_TRINITY_DN4855_c0_g1_i1p1
Bacterosira_constricta_CCMP991_TRINITY_DN18808_c0_g2_i1p1
AJA262-20_Thalassiosira_delicatula_WR59_00760-R1
AJA262-20_Thalassiosira_delicatula_WR59_00761-R1
RCC2560_Thalassiosira_delicatula_WR37_03721-R1
AJA262-20_Thalassiosira_delicatula_WR59_05004-R1
RCC2560_Thalassiosira_delicatula_WR37_00255-R1
Thalassiosira_rotula_GSO102_TRINITY_DN11330_c1_g1_i1p1
Thalassiosira_rotula_CCMP3096_TRINITY_DN13016_c0_g2_i1p1
Thalassiosira_rotula_GSO102_TRINITY_DN11330_c0_g1_i1p1
Thalassiosira_rotula_CCMP3096_TRINITY_DN13016_c0_g1_i1p1
Thalassiosira_gravida_GMp14c1_TRINITY_DN12441_c1_g1_i1p1
AJA294-31_Shionodiscus_oestrupii_WR58_05837-R1
Thalassiosira_antarctica_CCMP982_TRINITY_DN8687_c2_g1_i1p1
Thalassiosira_minuscula_CCMP1093_TRINITY_DN21186_c1_g1_i1p1
Thalassiosira_minuscula_CCMP1093_TRINITY_DN21186_c0_g1_i1p1
AJA294-31_Shionodiscus_oestrupii_WR58_00809-R1
Thalassiosira_minuscula_CCMP1093_TRINITY_DN21186_c3_g1_i1p1
Thalassiosira_antarctica_CCMP982_TRINITY_DN8687_c0_g1_i1p1
Thalassiosira_sp_NH16_TRINITY_DN7449_c1_g1_i1p1
CCMP995_Detonula_confervacea_WR97_08355-R1
CCMP975_Detonula_confervacea_WR67_01765-R1
CCMP995_Detonula_confervacea_WR97_08214-R1
AJA261-08_Thalassiosira_pacifica_WR39_00332-R3
Thalassiosira_pacifica_CCMP989_TRINITY_DN13609_c0_g2_i2p1
Thalassiosira_pacifica_CCMP989_TRINITY_DN13609_c0_g1_i1p1
Thalassiosira_sp_NH16_TRINITY_DN6726_c5_g1_i1p1
AJA248-18_Thalassiosira_sp_WR36_00303-R1
Thalassiosira_sp_NH16_TRINITY_DN6726_c0_g1_i1p1
AJA248-14_Thalassiosira_exigua_WR44_00073-R2
AJA257-08_Thalassiosira_mediterranea_WR41_00076-R1
CCMP1005_Thalassiosira_oceanica_Thaoce1_73930
tho15560
ECT2AJA-044_Thalassiosira_profunda_TPROF_003128-RA
CCMP1005_Thalassiosira_oceanica_Thaoce1_73931
AJA358-04_Skeletonema_tropicum_WR35_04846-R1
AJA274-08_Skeletonema_grethae_WR34_00360-R1
Skeletonema_japonicum_CCMP2506_TRINITY_DN8060_c2_g1_i1p1
Skeletonema_japonicum_CCMP2506_TRINITY_DN8060_c1_g1_i1p1
Skeletonema_japonicum_CCMP2506_TRINITY_DN8060_c0_g1_i1p1
Skeletonema_menzelii_CCMP793_TRINITY_DN5571_c2_g1_i1p1
AJA215-34_Skeletonema_cf_menzelii_WR26_08593-R1
Skeletonema_menzelii_CCMP793_TRINITY_DN5571_c0_g1_i1p1
Skeletonema_menzelii_CCMP793_TRINITY_DN5571_c3_g1_i1p1
Skeletonema_costatum_1716_TRINITY_DN828_c0_g1_i1p1
AJA304-46_Skeletonema_dohrnii_WR65_00408-R1
Skeletonema_costatum_1716_TRINITY_DN2329_c0_g1_i1p1
AJA304-46_Skeletonema_dohrnii_WR65_00858-R1
Skeletonema_marinoi_SM1012Hels-07_TRINITY_DN4885_c0_g1_i1p1
Skeletonema_costatum_1716_TRINITY_DN2163_c0_g1_i1p1
Scheldt_g50_i0
AJA081-3_Skeletonema_potamos_WR27_03353-R1
AJA131-32_Mediolabrus_comicus_WR94_00864-R1
Conticribra_weissflogii_CCMP1010_TRINITY_DN11027_c1_g2_i1p1
Eunotogramma_lunatum_ECT2AJA-043_TRINITY_DN16965_c0_g3_i1
AJA299-11_Cyclotella_atomus_WR98_05065-R1
AJA232-09_Cyclotella_atomus_WR45_02987-R1
AJA010-31_Cyclotella_atomus_WR22_03467-R1
Cyclotella_atomus_AJA264-12_TRINITY_DN59048_c0_g1_i1p1
AJA297-15_Cyclotella_baltica_WR54_11021-R1
Cyclotella_sp_CCMP338_TRINITY_DN9611_c5_g1_i1p1
AJA259-04_Cyclotella_choctawhatcheeana_WR53_00749-R1
AJA297-15_Cyclotella_baltica_WR54_05169-R1
Scheldt_g9_i0
AJA164-11_Cyclotella_cf_meneghiniana_WR24_01333-R2
AJA178-1_Cyclotella_meneghiniana_WR30_00633-R1
AJA299-14_Cyclotella_meneghiniana_WR31_06953-R1
AJA299-14_Cyclotella_meneghiniana_WR31_03093-R1
CCMP332_Cyclotella_cryptica_CCRYP_000457-RB
CC00G202730
AJA263-27_Cyclotella_cryptica_WR18_02676-R1
AJA349-1_Cyclotella_distinguenda_WR73_00361-R1
AJA303-46_Cyclotella_kingstonii_WR75_00279-R1
Cyclotella_sp_FW_TRINITY_DN7023_c9_g1_i1p1
Cyclotella_sp_FW_TRINITY_DN7023_c6_g1_i1p1
CCMP1335_Cyclotella_nana_XP_002288087_1
tps104840
Cyclotella_sp_FW_TRINITY_DN7023_c7_g1_i1p1
Cyclotella_sp_FW_TRINITY_DN7023_c3_g1_i1p1
Cyclotella_sp_FW_TRINITY_DN7023_c0_g1_i1p1
Cyclotella_sp_FW_TRINITY_DN7023_c5_g1_i1p1
Cyclotella_sp_FW_TRINITY_DN7023_c1_g1_i1p1
Eunotogramma_lunatum_ECT2AJA-043_TRINITY_DN32268_c0_g2_i1
Conticribra_weissflogii_CCMP1010_TRINITY_DN11027_c8_g1_i1p1
Scheldt_g195_i0
AJA302-05_Thalassiosira_livingstoniorum_WR56_00777-R1
Conticribra_weissflogii_CCMP1010_TRINITY_DN11027_c8_g2_i1p1
AJA158-1_Conticribra_weissflogii_WR49_01619-R1
CCMP988_Conticribra_guillardii_WR38_01127-R1
RCC2039_Porosira_glacialis_WR100_03451-R1
Lauderia_annulata_RCC5796_TRINITY_DN14047_c0_g3_i1p1
RCC2039_Porosira_glacialis_WR100_05193-R1
Lauderia_annulata_RCC5796_TRINITY_DN14047_c0_g1_i1p1
Lauderia_annulata_RCC5796_TRINITY_DN14047_c0_g4_i1p1
Ditylum_brightwellii_GSO105_TRINITY_DN11835_c3_g1_i1p1
Eunotogramma_lunatum_ECT2AJA-043_TRINITY_DN32268_c0_g1_i1
Ditylum_brightwellii_GSO105_TRINITY_DN11835_c2_g1_i1p1
Scheldt_g259593_i0
Scheldt_g308_i0
Scheldt_g308_i1
Lithodesmium_intricatum_ECT2AJA-029_TRINITY_DN23938_c1_g4_i1
Ditylum_brightwellii_GSO105_TRINITY_DN11835_c0_g1_i1p1
MMETSP0321-DN11382_c3_g2_i1.p1
Sample
Taxonomic clade
Thalassiosira clade
Cyclotella clade
Skeletonema clade
Conticribra clade
Porosira clade
Pennate diatoms
Normalized CPM
1.00
0.75
0.50
0.25
0.00
Maximal expression
No expression
Differential expression
Upregulated
Not DE
Downregulated
Reference gene

Supplementary Figure 20: Cladogram of midpoint-rooted bootstrap consensus phylogenetic tree of DNAH5/8 protein sequences from the Scheldt microcosm metatranscriptome as well as reference DNAH5/8 proteins from Roberts et al. (2023), Pinder et al., in prep., Audoor et al. (2024) and the PLAZA Diatoms v1.0 platform (Osuna-Cruz et al. 2020). Clades within the Thalassiosirales follow Roberts et al. (2023). Next to the phylogeny, a heatmap shows the expression of Scheldt transcripts as normalized counts per million (CPM) in control (0 ppt) and salt-treated (10 ppt) conditions. Ppt = parts per thousand.

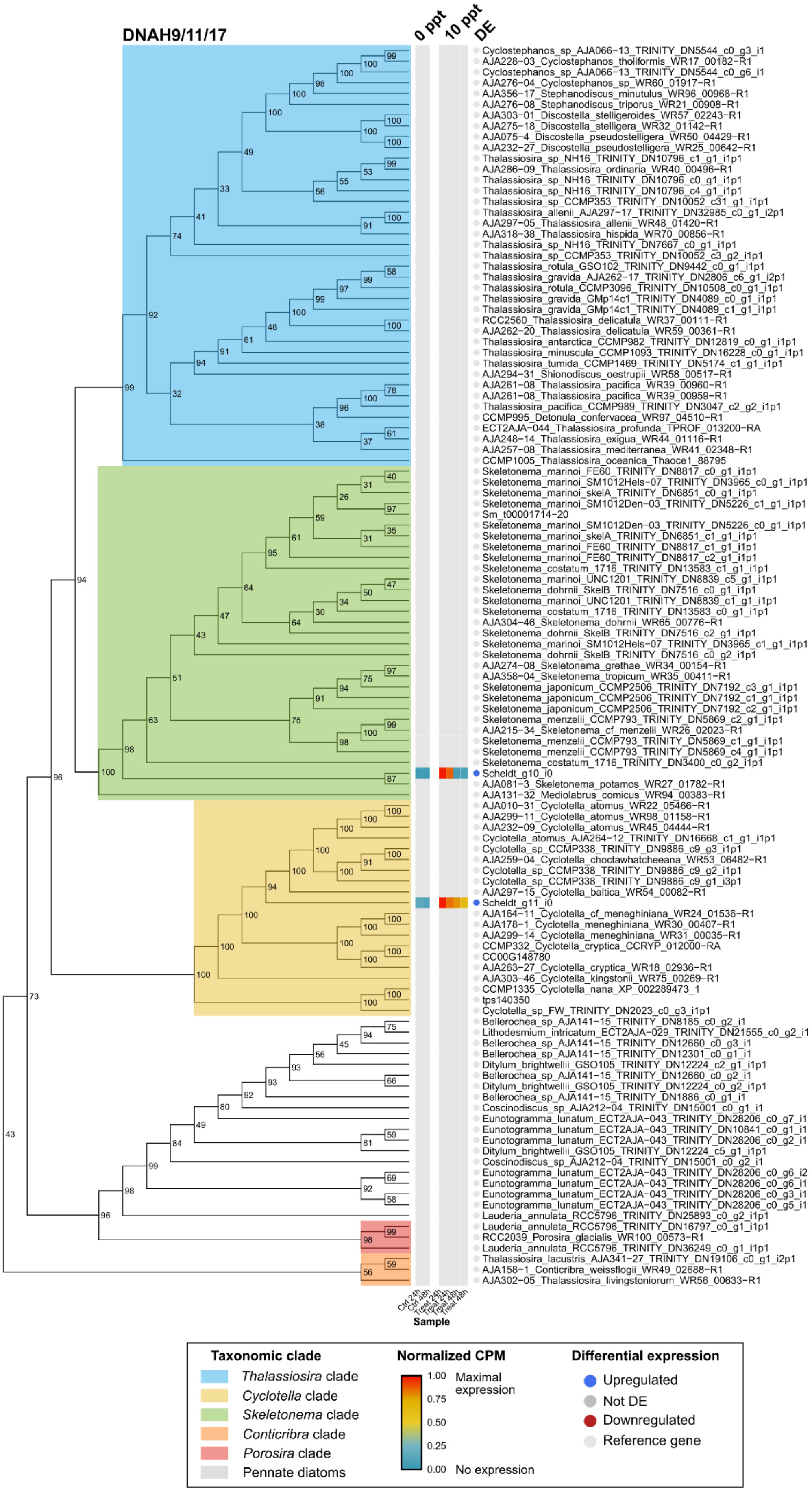
DNAH9/11/17
0 ppt
10 ppt
DE
Cyclostephanos_sp_AJA066−13_TRINITY_DN5544_c0_g3_i1
AJA228−03_Cyclostephanos_tholiformis_WR17_00182−R1
Cyclostephanos_sp_AJA066−13_TRINITY_DN5544_c0_g6_i1
AJA276−04_Cyclostephanos_sp_WR60_01917−R1
AJA356−17_Stephanodiscus_minutulus_WR96_00968−R1
AJA276−08_Stephanodiscus_triporus_WR21_00908−R1
AJA303−01_Discostella_stelligeroides_WR57_02243−R1
AJA275−18_Discostella_stelligera_WR32_01142−R1
AJA075−4_Discostella_pseudostelligera_WR50_04429−R1
AJA232−27_Discostella_pseudostelligera_WR25_00642−R1
Thalassiosira_sp_NH16_TRINITY_DN10796_c1_g1_i1p1
AJA286−09_Thalassiosira_ordinaria_WR40_00496−R1
Thalassiosira_sp_NH16_TRINITY_DN10796_c0_g1_i1p1
Thalassiosira_sp_NH16_TRINITY_DN10796_c4_g1_i1p1
Thalassiosira_sp_CCMP353_TRINITY_DN10052_c31_g1_i1p1
Thalassiosira_allenii_AJA297−17_TRINITY_DN32985_c0_g1_i2p1
AJA297−05_Thalassiosira_allenii_WR48_01420−R1
AJA318−38_Thalassiosira_hispida_WR70_00856−R1
Thalassiosira_sp_NH16_TRINITY_DN7667_c0_g1_i1p1
Thalassiosira_sp_CCMP353_TRINITY_DN10052_c3_g2_i1p1
Thalassiosira_rotula_GSO102_TRINITY_DN9442_c0_g1_i1p1
Thalassiosira_gravida_AJA262−17_TRINITY_DN2806_c6_g1_i2p1
Thalassiosira_rotula_CCMP3096_TRINITY_DN10508_c0_g1_i1p1
Thalassiosira_gravida_GMp14c1_TRINITY_DN4089_c0_g1_i1p1
Thalassiosira_gravida_GMp14c1_TRINITY_DN4089_c1_g1_i1p1
RCC2560_Thalassiosira_delicatula_WR37_00111−R1
AJA262−20_Thalassiosira_delicatula_WR59_00361−R1
Thalassiosira_antarctica_CCMP982_TRINITY_DN12819_c0_g1_i1p1
Thalassiosira_minuscula_CCMP1093_TRINITY_DN16228_c0_g1_i1p1
Thalassiosira_tumida_CCMP1469_TRINITY_DN5174_c1_g1_i1p1
AJA294−31_Shionodiscus_oestrupii_WR58_00517−R1
AJA261−08_Thalassiosira_pacifica_WR39_00960−R1
AJA261−08_Thalassiosira_pacifica_WR39_00959−R1
Thalassiosira_pacifica_CCMP989_TRINITY_DN3047_c2_g2_i1p1
CCMP995_Detonula_confervacea_WR97_04510−R1
ECT2AJA−044_Thalassiosira_profunda_TPROF_013200−RA
AJA248−14_Thalassiosira_exigua_WR44_01116−R1
AJA257−08_Thalassiosira_mediterranea_WR41_02348−R1
CCMP1005_Thalassiosira_oceanica_Thaoce1_88795
Skeletonema_marinoi_FE60_TRINITY_DN8817_c0_g1_i1p1
Skeletonema_marinoi_SM1012Hels−07_TRINITY_DN3965_c0_g1_i1p1
Skeletonema_marinoi_skelA_TRINITY_DN6851_c0_g1_i1p1
Skeletonema_marinoi_SM1012Den−03_TRINITY_DN5226_c1_g1_i1p1
Sm_t00001714−20
Skeletonema_marinoi_SM1012Den−03_TRINITY_DN5226_c0_g1_i1p1
Skeletonema_marinoi_skelA_TRINITY_DN6851_c1_g1_i1p1
Skeletonema_marinoi_FE60_TRINITY_DN8817_c1_g1_i1p1
Skeletonema_marinoi_FE60_TRINITY_DN8817_c2_g1_i1p1
Skeletonema_costatum_1716_TRINITY_DN13583_c1_g1_i1p1
Skeletonema_marinoi_UNC1201_TRINITY_DN8839_c5_g1_i1p1
Skeletonema_dohrnii_SkelB_TRINITY_DN7516_c0_g1_i1p1
Skeletonema_marinoi_UNC1201_TRINITY_DN8839_c1_g1_i1p1
Skeletonema_costatum_1716_TRINITY_DN13583_c0_g1_i1p1
AJA304−46_Skeletonema_dohrnii_WR65_00776−R1
Skeletonema_dohrnii_SkelB_TRINITY_DN7516_c2_g1_i1p1
Skeletonema_marinoi_SM1012Hels−07_TRINITY_DN3965_c1_g1_i1p1
Skeletonema_dohrnii_SkelB_TRINITY_DN7516_c0_g2_i1p1
AJA274−08_Skeletonema_grethae_WR34_00154−R1
AJA358−04_Skeletonema_tropicum_WR35_00411−R1
Skeletonema_japonicum_CCMP2506_TRINITY_DN7192_c3_g1_i1p1
Skeletonema_japonicum_CCMP2506_TRINITY_DN7192_c1_g1_i1p1
Skeletonema_japonicum_CCMP2506_TRINITY_DN7192_c2_g1_i1p1
Skeletonema_menzelii_CCMP793_TRINITY_DN5869_c2_g1_i1p1
AJA215−34_Skeletonema_cf_menzelii_WR26_02023−R1
Skeletonema_menzelii_CCMP793_TRINITY_DN5869_c1_g1_i1p1
Skeletonema_menzelii_CCMP793_TRINITY_DN5869_c4_g1_i1p1
Skeletonema_costatum_1716_TRINITY_DN3400_c0_g2_i1p1
Scheldt_g10_i0
AJA081−3_Skeletonema_potamos_WR27_01782−R1
AJA131−32_Mediolabrus_comicus_WR94_00383−R1
AJA010−31_Cyclotella_atomus_WR22_05466−R1
AJA299−11_Cyclotella_atomus_WR98_01158−R1
AJA232−09_Cyclotella_atomus_WR45_04444−R1
Cyclotella_atomus_AJA264−12_TRINITY_DN16668_c1_g1_i1p1
Cyclotella_sp_CCMP338_TRINITY_DN9886_c9_g3_i1p1
AJA259−04_Cyclotella_choctawhatcheeana_WR53_06482−R1
Cyclotella_sp_CCMP338_TRINITY_DN9886_c9_g2_i1p1
Cyclotella_sp_CCMP338_TRINITY_DN9886_c9_g1_i3p1
AJA297−15_Cyclotella_baltica_WR54_00082−R1
Scheldt_g11_i0
AJA164−11_Cyclotella_cf_meneghiniana_WR24_01536−R1
AJA178−1_Cyclotella_meneghiniana_WR30_00407−R1
AJA299−14_Cyclotella_meneghiniana_WR31_00035−R1
CCMP332_Cyclotella_cryptica_CCRYP_012000−RA
CC00G148780
AJA263−27_Cyclotella_cryptica_WR18_02936−R1
AJA303−46_Cyclotella_kingstonii_WR75_00269−R1
CCMP1335_Cyclotella_nana_XP_002289473_1
tps140350
Cyclotella_sp_FW_TRINITY_DN2023_c0_g3_i1p1
Bellerochea_sp_AJA141−15_TRINITY_DN8185_c0_g2_i1
Lithodesmium_intricatum_ECT2AJA−029_TRINITY_DN21555_c0_g2_i1
Bellerochea_sp_AJA141−15_TRINITY_DN12660_c0_g3_i1
Bellerochea_sp_AJA141−15_TRINITY_DN12301_c0_g1_i1
Ditylum_brightwellii_GSO105_TRINITY_DN12224_c2_g1_i1p1
Bellerochea_sp_AJA141−15_TRINITY_DN12660_c0_g2_i1
Ditylum_brightwellii_GSO105_TRINITY_DN12224_c0_g2_i1p1
Bellerochea_sp_AJA141−15_TRINITY_DN1886_c0_g1_i1
Coscinodiscus_sp_AJA212−04_TRINITY_DN15001_c0_g1_i1
Eunotogramma_lunatum_ECT2AJA−043_TRINITY_DN28206_c0_g7_i1
Eunotogramma_lunatum_ECT2AJA−043_TRINITY_DN10841_c0_g1_i1
Eunotogramma_lunatum_ECT2AJA−043_TRINITY_DN28206_c0_g2_i1
Ditylum_brightwellii_GSO105_TRINITY_DN12224_c5_g1_i1p1
Coscinodiscus_sp_AJA212−04_TRINITY_DN15001_c0_g2_i1
Eunotogramma_lunatum_ECT2AJA−043_TRINITY_DN28206_c0_g6_i2
Eunotogramma_lunatum_ECT2AJA−043_TRINITY_DN28206_c0_g6_i1
Eunotogramma_lunatum_ECT2AJA−043_TRINITY_DN28206_c0_g3_i1
Eunotogramma_lunatum_ECT2AJA−043_TRINITY_DN28206_c0_g5_i1
Lauderia_annulata_RCC5796_TRINITY_DN25893_c0_g2_i1p1
Lauderia_annulata_RCC5796_TRINITY_DN16797_c0_g1_i1p1
RCC2039_Porosira_glacialis_WR100_00573−R1
Lauderia_annulata_RCC5796_TRINITY_DN36249_c0_g1_i1p1
Thalassiosira_lacustris_AJA341−27_TRINITY_DN19106_c0_g1_i2p1
AJA158−1_Conticribra_weissflogii_WR49_02688−R1
AJA302−05_Thalassiosira_livingstoniorum_WR56_00633−R1
Ctrl 24h
Ctrl 48h
Treat 24h
Treat 48h
Treat 24h
Treat 48h
Sample
Taxonomic clade
Thalassiosira clade
Cyclotella clade
Skeletonema clade
Conticribra clade
Porosira clade
Pennate diatoms
Normalized CPM
1.00
0.75
0.50
0.25
0.00
Maximal expression
No expression
Differential expression
Upregulated
Not DE
Downregulated
Reference gene

Supplementary Figure 21: Cladogram of midpoint-rooted bootstrap consensus phylogenetic tree of DNAH9/11/17 protein sequences from the Scheldt microcosm metatranscriptome as well as reference DNAH9/11/17 proteins from Roberts et al. (2023), Pinder et al., in prep., Audoor et al. (2024) and the PLAZA Diatoms v1.0 platform (Osuna-Cruz et al. 2020). Clades within the Thalassiosirales follow Roberts et al. (2023). Next to the phylogeny, a heatmap shows the expression of Scheldt transcripts as normalized counts per million (CPM) in control (0 ppt) and salt-treated (10 ppt) conditions. Ppt = parts per thousand.

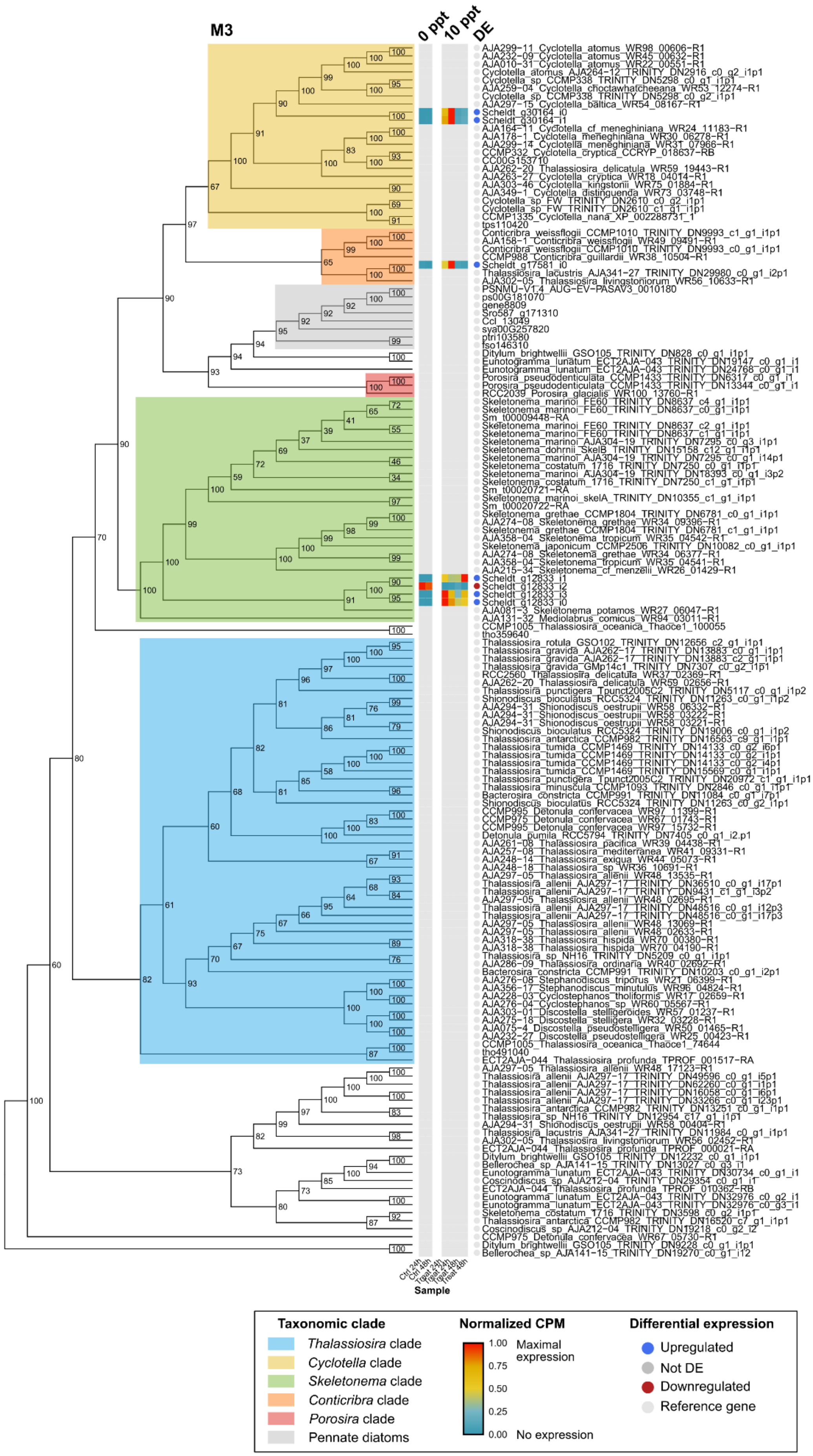
M3
0 ppt
10 ppt
DE
Sample
Taxonomic clade
Thalassiosira clade
Cyclotella clade
Skeletonema clade
Conticribra clade
Porosira clade
Pennate diatoms
Normalized CPM
1.00
0.75
0.50
0.25
0.00
Maximal expression
No expression
Differential expression
Upregulated
Not DE
Downregulated
Reference gene

Supplementary Figure 22: Cladogram of midpoint-rooted bootstrap consensus phylogenetic tree of M3 protein sequences from the Scheldt microcosm metatranscriptome as well as reference M3 proteins from Roberts et al. (2023), Audoor et al. (2024) and the PLAZA Diatoms v1.0 platform (Osuna-Cruz et al. 2020). Clades within the Thalassiosirales follow Roberts et al. (2023). Next to the phylogeny, a heatmap shows the expression of Scheldt transcripts as normalized counts per million (CPM) in control (0 ppt) and salt-treated (10 ppt) conditions. Ppt = parts per thousand.

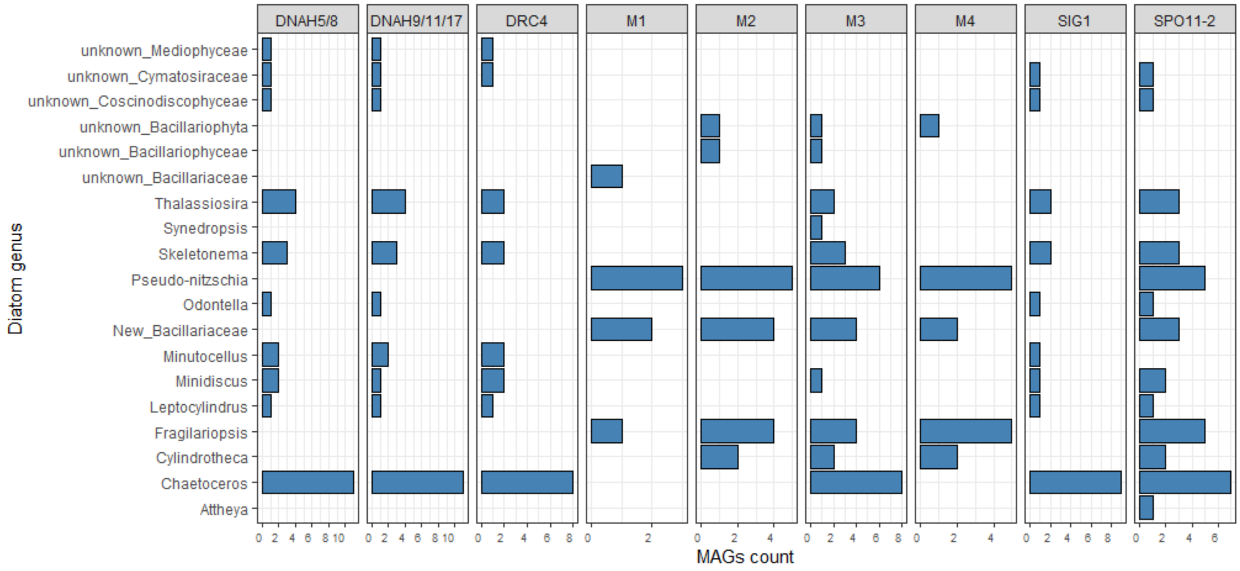

Supplementary Figure 23: Bar plots showing taxonomic annotation and number of diatom MAGs that encode each marker. The number of MAG hits that were phylogenetically positioned within the original clade of reference marker proteins ("phylogenetic selection") is reported for each diatom genus.

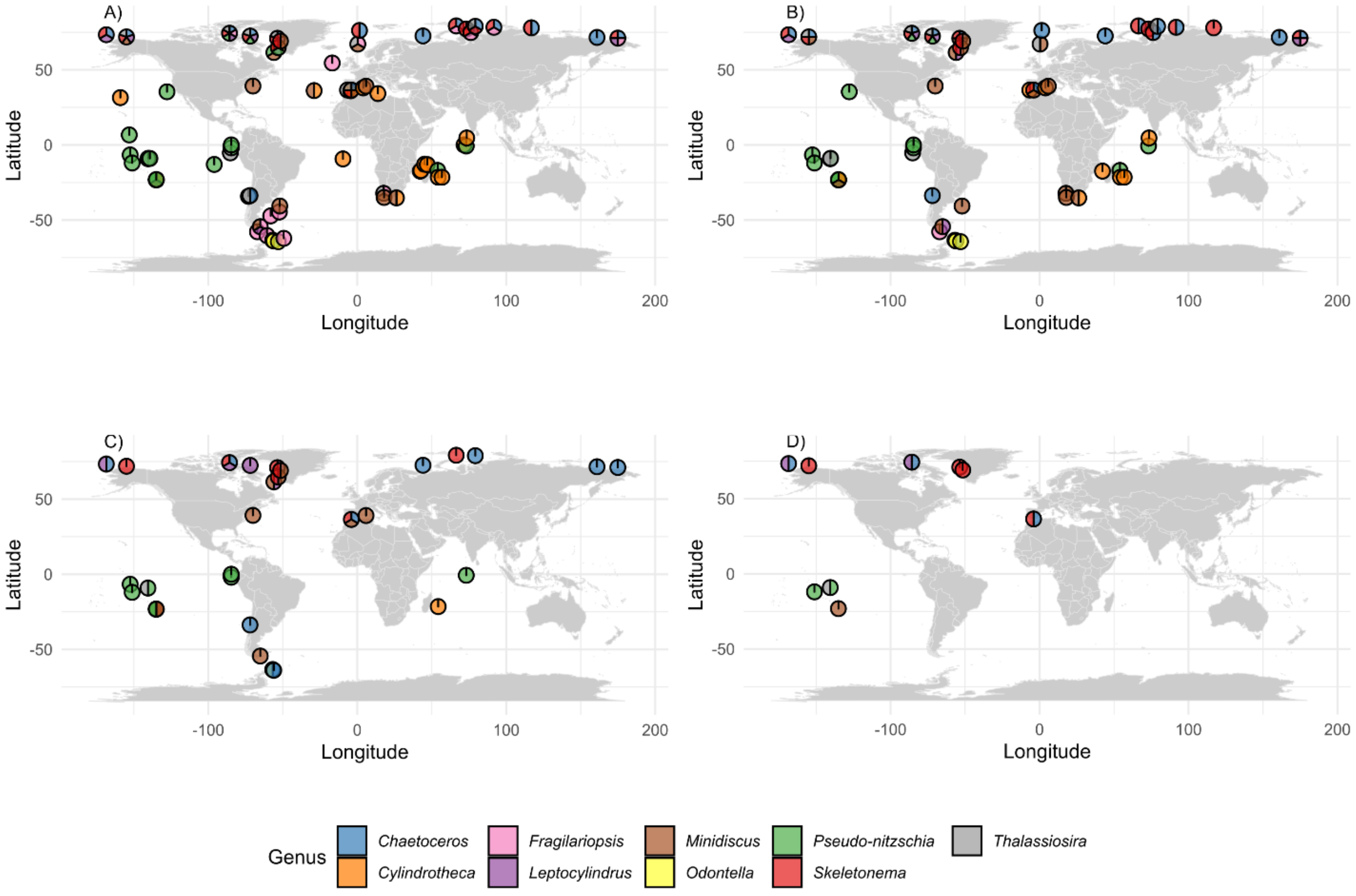

Supplementary Figure 24. Biogeography of MAGs co-expressing sexual markers, with progressively increasing numbers of markers. World map showing *Tara* stations where one or more Metagenome-Assembled Genomes (MAGs) co-express the positive control gene *SPO11-2*, along with at least (A) one, (B) two, (C) three, and (D) four additional sexual markers. Both *SPO11-2* and the sexual markers are considered expressed if they surpass the defined transcripts per million (TPM) thresholds and have at least two reads. The pie charts indicate the genera co-expressing the respective markers at each station, where colors represent the genus-level assignment of MAGs.

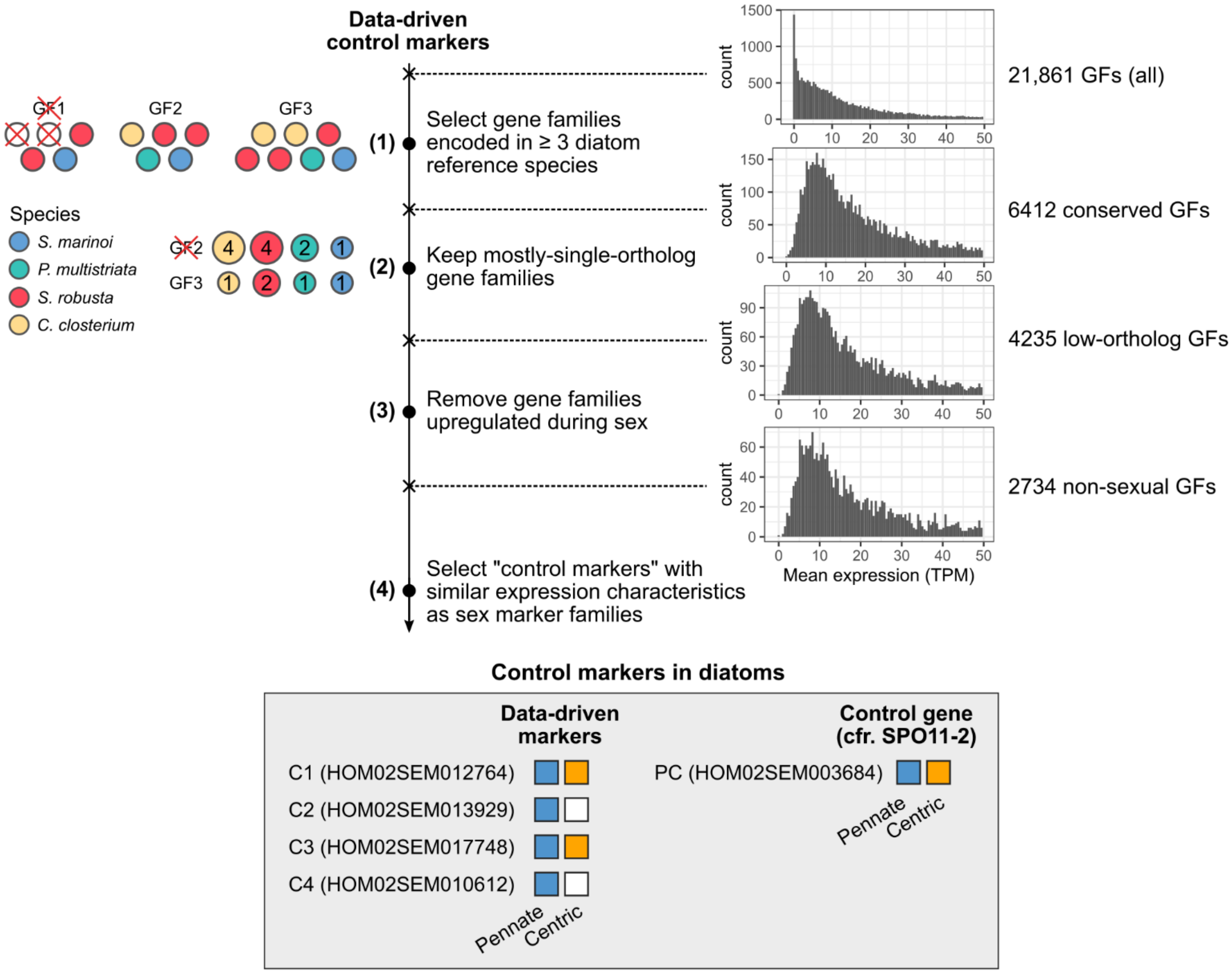

Supplementary Figure 25: Bioinformatics pipeline for the discovery of “C1-C4” control marker families with similar characteristics as the data-driven sex markers M1-M4, and “PC” similar to the positive control gene SPO11-2. GFs: gene families.

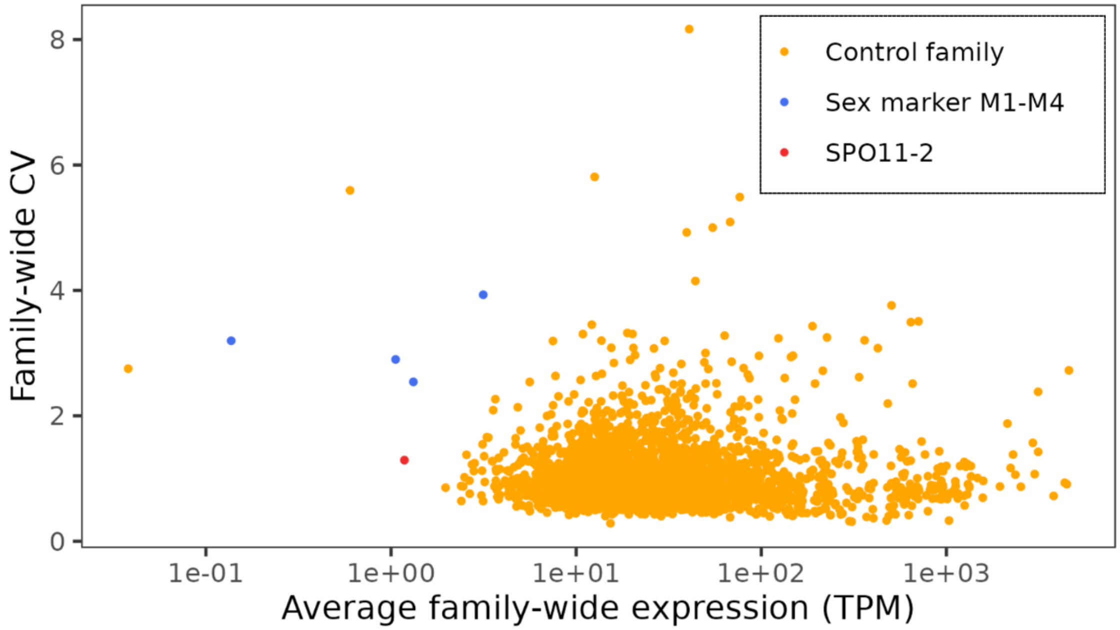

Supplementary Figure 26: Scatterplot showing the coefficient of variation (CV) and average expression (transcripts per million, TPM, note the logarithmic scale). Each point represents a single gene family. Dots are coloured by type: potential control family (orange), data-driven sex markers (blue) and positive control gene SPO11-2 (red). Averages and CV were calculated including all gene family homologs that belong to any of the following four species: *Pseudo-nitzschia multistriata, Seminavis robusta, Skeletonema marinoi,* and *Cylindrotheca closterium*. Expression statistics of sex markers were calculated for vegetative conditions only (non-sexual samples), while for control families they were calculated over all conditions (non-sexual and sexual).

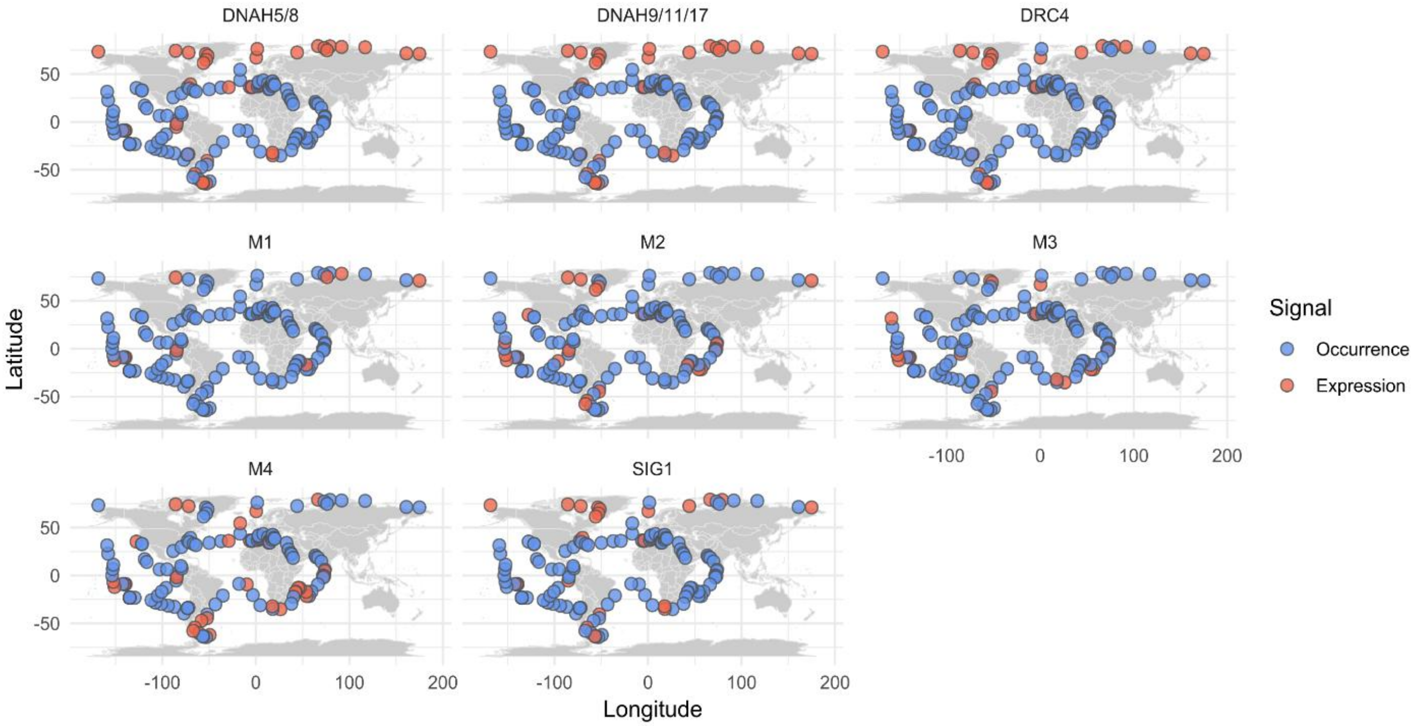

Supplementary Figure 27: Occurrence at MAG level and expression of the selected genes across stations. Global-scale representation of the occurrence at the genome level (blue dots) and expression signal (red dots) for each of the marker genes separately. Sexual markers are considered expressed if they surpass the defined TPM (transcripts per million) thresholds and have at least two reads.

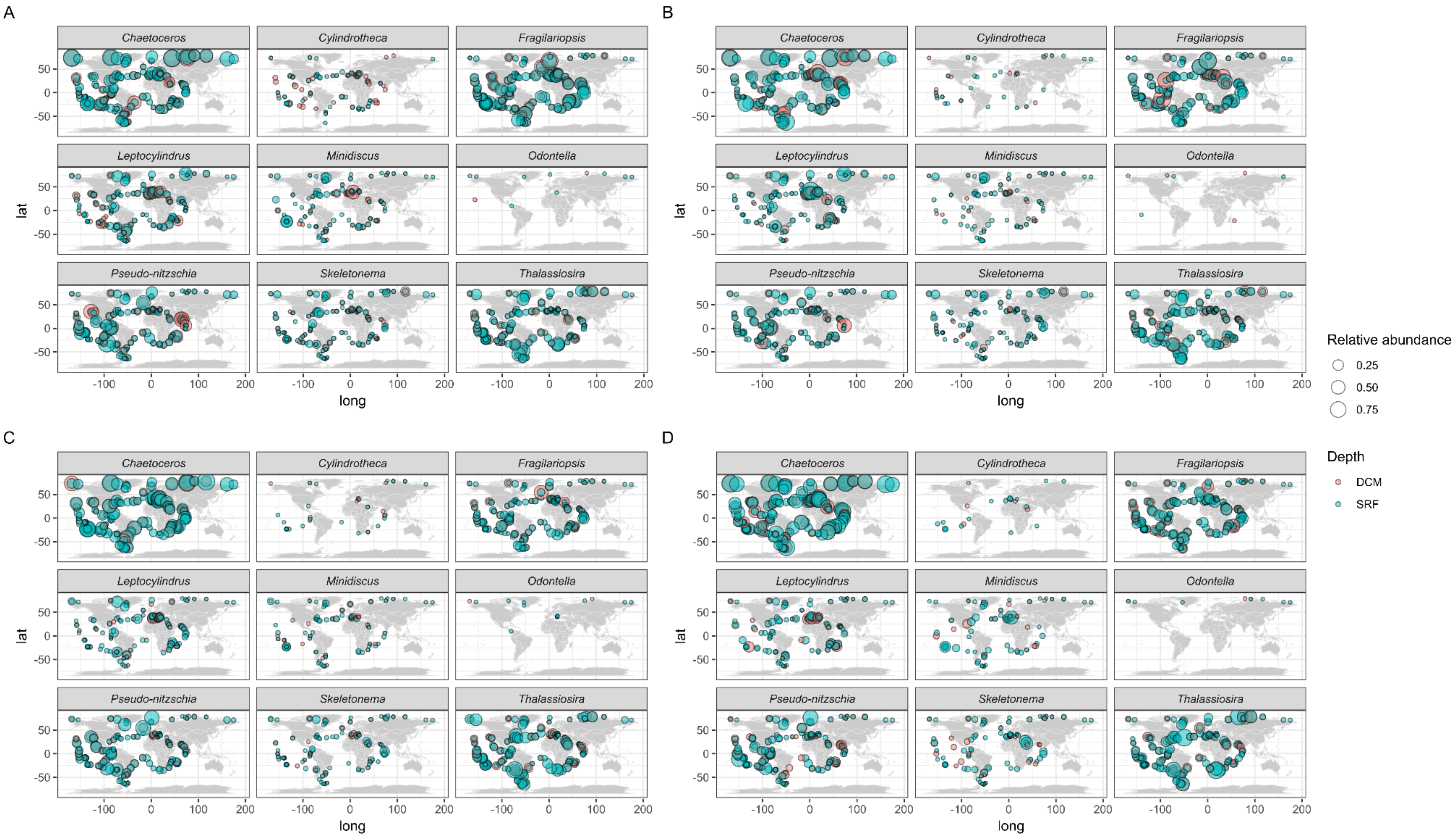
A
B
C
D
Chaetoceros
Cylindrotheca
Fragilariopsis
Leptocylindrus
Minidiscus
Odontella
Pseudo-nitzschia
Skeletonema
Thalassiosira
lat
long
50
0
-50
-100
0
100
200
Relative abundance
0.25
0.50
0.75
Depth
DCM
SRF

Supplementary Figure 28: Global distribution and relative abundance of the selected diatom genera**.** Bubble areas represent the abundance of each genus relative to the total diatom abundance at each location, whereas the color represents the two sampled depths (DCM: Deep Chlorophyll Maximum; SRF: surface). The maps show the relative abundance in the A) pico; B) nano; C) micro and D) meso-planktonic size classes.

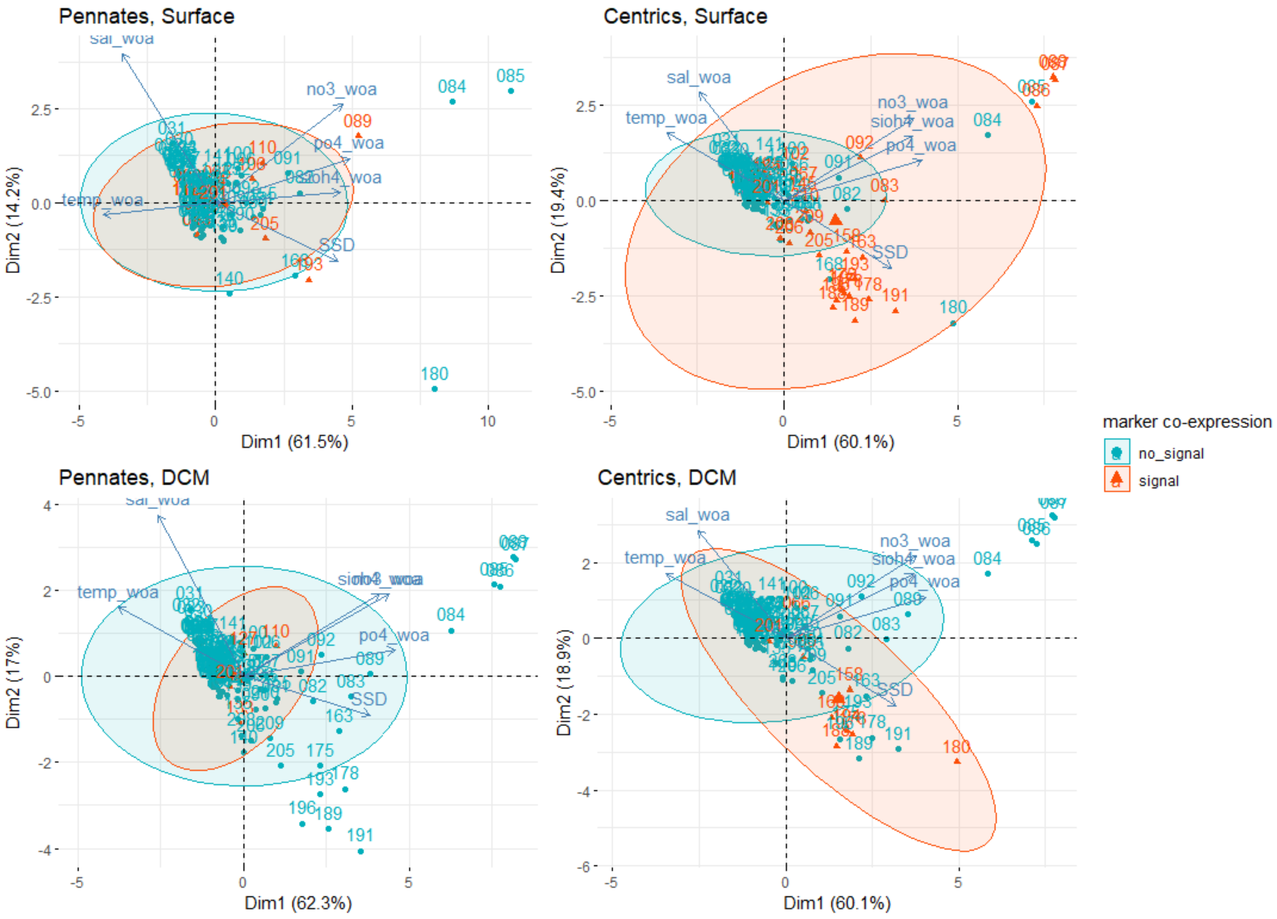

Supplementary Figure 29: Principal component analysis of environmental parameters from *Tara* Oceans stations with presence (red) or absence (blue) of sexual reproduction signals. Stations are grouped by color based on these signals. The analysis was done separately for Pennate and Centric diatoms and Surface and DCM (Deep Chlorophyll Maximum) depths. Parameters include temperature (temp_woa), salinity (sal_woa), nitrates (no3_woa), phosphates (po4_woa) and silicates (sioh_woa) from the World Ocean Atlas, and Sea Sunshine Duration (SSD) from the U.S. Naval Observatory.

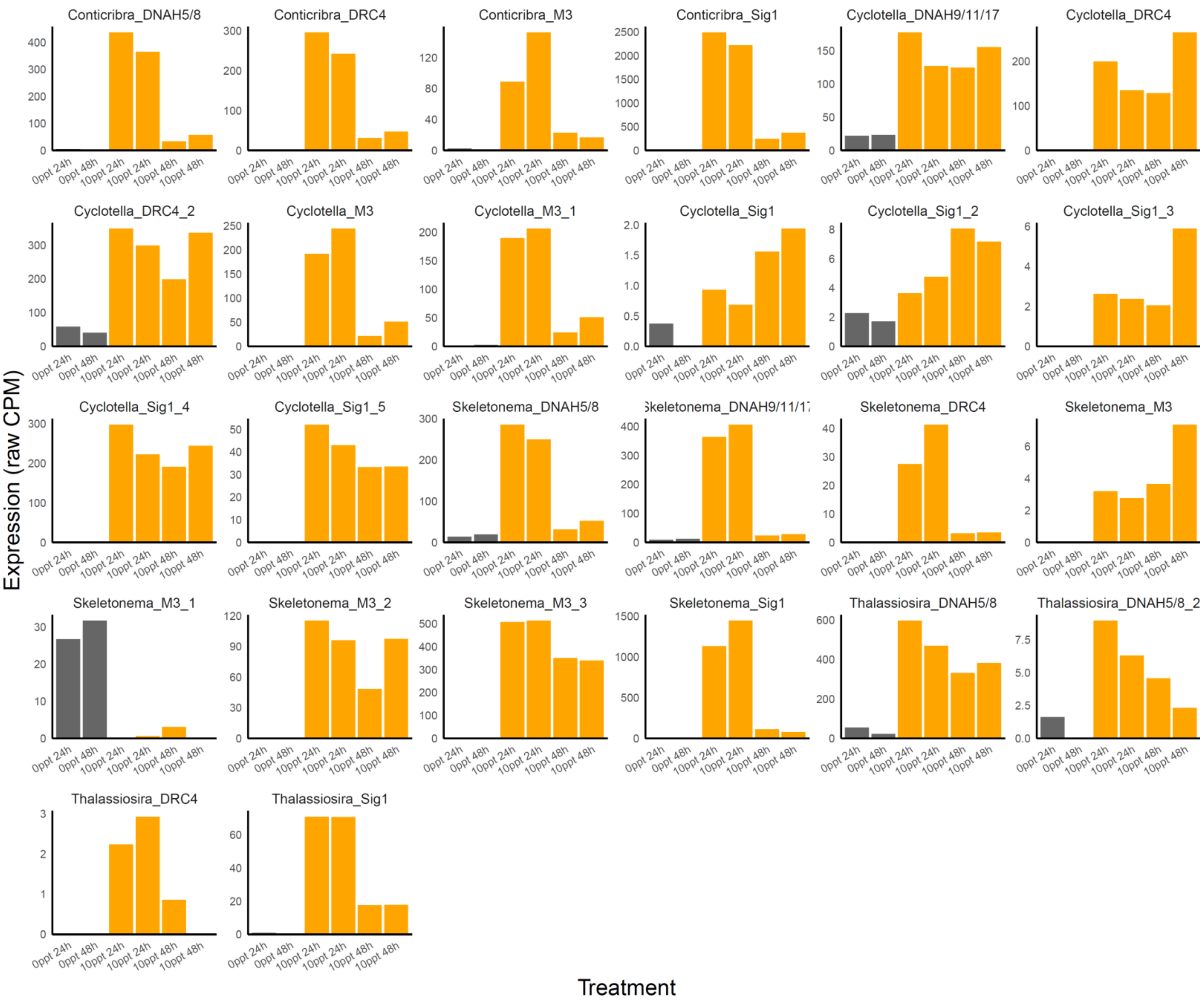

Supplementary Figure 30: Bar plots showing the expression (unscaled counts per million, CPM) of sex marker hits in microcosm metatranscriptome samples (x-axis). Ppt: parts per thousand. Bars belonging to untreated control samples are coloured in grey, sexualized samples using a salt treatment are coloured in orange.

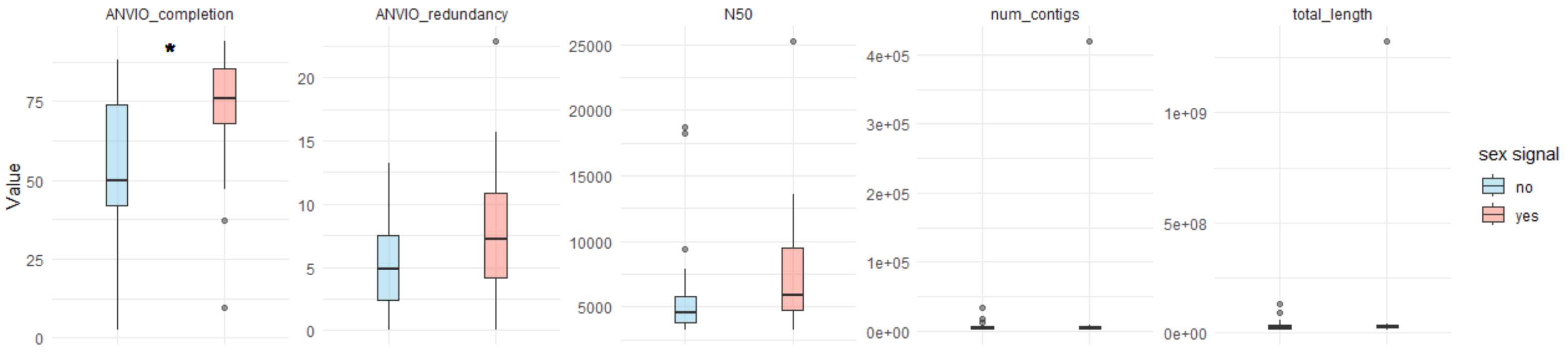

Supplementary Figure 31. Comparison of key assembly metrics for *Tara* Oceans diatom Metagenome-Assembled Genomes (MAGs). Box plots show the distributions of each metric, color-coded by whether a MAG exhibits a sexual reproduction signal (“yes”, red) or not (“no”, blue). Total_length: sum of all contig lengths in the MAG. N50: the contig length at which half of the total assembly length is contained in contigs of that length or longer. ANVIO_completion and ANVIO_redundancy: estimated completeness and redundancy from the Anvi’o pipeline. An asterisk indicates a significant difference between the two groups ($p < 0.05$ Mann-Whitney U-test with Bonferroni correction).

Supplementary Tables

Table S1: Overview of RNA-seq datasets included in the comparative analysis of sexual reproducing diatoms. *"#Ctrl" and "#Sex" depict the number of control and sexualized replicates respectively. SIP: sex inducing pheromone. Time points are assigned to their defining sexual cell stage as follows: S: pheromone signaling, P: gametangia, GZ: gametes and zygotes, A: auxospores. The sequencing libraries consist of either single-end (SE) or paired-end (PE) reads with the length (base pairs) of a single read indicated.*

Species	Stage	Treatment	Time	#Ctrl	#Sex	Library	Reference	Data Source
P. multistriata	S	Crossed	1h	3+3	3	SE50	Annunziata et al. (2022)	PRJEB37110
P. multistriata	GZ	Crossed	24h	3+3	3	SE50	Annunziata et al. (2022)	PRJEB37110
P. multistriata	A	Crossed	120h	3+3	3	SE50	Annunziata et al. (2022)	PRJEB37110
S. robusta	S-	SIP treated MT-	3h	3	3	PE150	Moeys et al. (2016)	PRJEB11784
S. robusta	S+	SIP treated MT+	3h	3	3	PE75	Bilcke et al. (2021)	PRJEB35793
S. robusta	P	Crossed	11h	3	3	PE75	Osuna-Cruz et al. (2020)	PRJEB36275
S. robusta	GZ	Crossed	14h	3	3	PE75	Osuna-Cruz et al. (2020)	PRJEB36275
S. robusta	A	Crossed	21h	3	2	PE75	Osuna-Cruz et al. (2020)	PRJEB36275
C. closterium	P	Crossed	9h	3	3	PE150	Audoor et al. (2024)	PRJEB49955
C. closterium	GZ	Crossed	14h	3	3	PE150	Audoor et al. (2024)	PRJEB49955
C. closterium	A	Crossed	27h	3	3	PE150	Audoor et al. (2024)	PRJEB49955
S. marinoi	P	Salinity-induced	40h	2	3	SE50	Ferrante et al. (2019)	PRJEB33171
S. marinoi	GZ	Salinity-induced	52h	2	3	SE50	Ferrante et al. (2019)	PRJEB33171

Table S2: Version number and data source for reference genome assembly and gene annotation files of the four species considered in this paper.

Species	Assembly	Annotation	Reference	Data source
P. multistriata	V1	Pm-1.4_mRNA_v3	Basu et al. (2017)	bioinformatics.psb.ugent.be/plaza/versions/plaza_diatoms_01/download<br>Original: bioinfo.szn.it/pmultistriata/
S. robusta	V1	Sro_v1.2 (mRNA)	Osuna-Cruz et al. (2020)	bioinformatics.psb.ugent.be/gdb/seminavis/Version1.2
C. closterium	V1	V1.2 (CDS)	Audoor et al. (2024)	bioinformatics.psb.ugent.be/orcae/overview/Cylcl
S. marinoi	V1.1.2	V1.1.2 (mRNA)	Pinder et al., in prep.	Gene models: Audoor et al. (2024)<br>NCBI: JATAAI000000000

__Table S3 Primer sequences used for the qPCR experiment.__ For each marker/reference gene, the Cylindrotheca closterium gene ID, gene family, forward and reverse primer sequences and melting temperature (Tm) are listed.

**Marker/reference gene**	**Cylindrotheca gene family (Audoor et al. 2024)**	***C. closterium* gene ID**	**Primer sequence (5'->3')**	**Tm (°C)**
**Marker 1:** Tubby-like	HOM338GF014279	Ccl_11047	CCGTATTGGGAAGCAAACAT	59.8
			TGGCTGAGAAAGGAATCCAC	60.2
**Marker 2:** Lectin-like	HOM338GF011666	Ccl_13876	GACGTGAGCGCACTATTGAA	60.0
			GGTTTGGTCCAAGAATCGAA	59.9
**Marker 3:** SIG/Hop2	HOM338GF004758	Ccl_13049	CGGAAGAAGACGAAGTCTGG	60.0
			CCAACCACCAAAGTCTTGCT	60.1
**Marker 4:** unknown	HOM338GF012301	Ccl_2381	CTCGGGCTCAAATTTATGGA	60.0
			AGCCAGCAAAAGAAGAACCA	60.0
**Histon 2B**	HOM338GF000129	Ccl_2393	GCCAAAACTCCAAGCAAGAA	60.4
			CATGATGGACATGCCCTTTT	60.7
**TATA box binding protein**	HOM338GF000610	Ccl_21857	ACTGTTGATGTGGGCTTTCC	60.0
			GTCGGACTCCTTCTTTGCAC	59.9
**β-tubulin**	HOM338GF000116	Ccl_5938	ACGGACGCTACTTGACTTGC	60.5
			CTCAAGTCCCTTTGGTGGAAC	60.9

***Table S4: Identification of meiotic markers in diatoms**. Homologs identified using blastp and phylogenic selection are indicated for S. marinoi, C. closterium, L. danicus and other centric diatoms in the PLAZA Diatoms platform (“PLAZA Diatoms homologs”). The corresponding PLAZA Diatoms Homologous gene family ID is also given.*

**Name**	**Source**	***S. marinoi***	***C. closterium***	***L. danicus* MMETSP**	**PLAZA Diatoms homologs**	**PLAZA Diatoms HOM02SEM GF**
Spo11-2	Patil et al. (2015)	Sm_t00007854-RA	Ccl_14394	MMETSP0321-DN9387_c1_g1_i1.p1	tho23880, tps204350, CC00G168580, ptri121280, fso158450, Sro229_g092990, sya00G147630, sya00G115100, gene20310, PSNMU-V1.4_AUG-EV-PASAV3_0108120, ps00G66820	000835
Mer3	Patil et al. (2015)	Sm_t00003921-RA	Ccl_4864	MMETSP0321-DN7074_c0_g1_i1.p1	CC00G09270, sya00G242600, PSNMU-V1.4_AUG-EV-PASAV3_0087420, ps00G101060, gene17732, gene14483, Sro155_g070300, ptri41950, fso181180, fso182260	000294
Mnd1	Patil et al. (2015)	Sm_t00010572-RA	Ccl_1830	MMETSP0321-DN3408_c0_g1_i1.p1 MMETSP0322-DN9167_c0_g1_i1.p2	tho166760, tps225900, CC00G116780, sya00G56160, fso111130, fso166330, PSNMU-V1.4_AUG-EV-PASAV3_0080640, ps00G95270, gene1640, Sro2127_g315750	003194
Msh4	Patil et al. (2015)	Sm_t00019914-RA	Ccl_4147	MMETSP1362-DN4073_c0_g2_i1.p1 MMETSP0321-DN10013_c6_g1_i1.p1	tho39680, tps93880, CC00G61950, ptri149960, fso03340, fso201680, sya00G74240, ps00G09760, Sro552_g165070	000161
Msh5	Patil et al. (2015)	Sm_t00007132-RA	Ccl_6699	MMETSP0321-DN9949_c0_g1_i2.p2	tho61620, tps02320, CC00G136100, fso184750, fso180160, ptri103220, Sro2738_g335960, ps00G191920, gene16407, PSNMU-V1.4_AUG-EV-PASAV3_0023810	000161
Rad51-A	Annunziata et al. (2022)	Sm_t00003409-RA	Ccl_19362	MMETSP1362-DN10217_c0_g1_i1.p1 MMETSP0322-DN993_c0_g1_i1.p1	CC00G202830, sya00G92960, ptri128550, fso191100, fso71610, Sro1748_g295150, PSNMU-V1.4_AUG-EV-PASAV3_0056780	000468

Table S5: Identification of flagella markers in centric diatoms*. For each gene class, the source of the query sequence ("Source") is given, as well as the Trinity gene identifier for the Ferrante et al. (2019) query genes ("S. marinoi Trinity"). Homologs identified using blastp and phylogenic selection are indicated for S. marinoi ("S. marinoi genome"), L. danicus ("L. danicus MMETSP") and other centric diatoms in the PLAZA Diatoms platform ("PLAZA diatom homologs"). The corresponding PLAZA Diatoms Homologous gene family ID is also given. As part of the selection procedure, the adjusted P-value of the "overall" contrast of our differential expression analysis is given. "Filtered" genes showed a too low expression level and were removed from the differential expression analysis. For DNAH9/11/17 (indicated with an asterisk), seven sequential partial genes from the S. marinoi genome were found that together make up the full gene. This gene was manually curated for protein prediction, HMM profile generation and phylogenetic analysis.*

**Name**	**Source**	***S. marinoi* Trinity**	***S. marinoi* genome**	**AdjP *S. marinoi***	***L. danicus* MMETSP**	**PLAZA Diatoms homologs**	**PLAZA Diatoms HOM02SEM GF**
Sig1	Armbrust (1999)	ND	Sm_t00002153-RA	3.50E-08	Missing	tps57670, CC00G180690, CC00G98110, CC00G27060	010699
Sig2	Armbrust (1999)	ND	*Missing*	*missing*	MMETSP0321-DN6305_c0_g1_i1.p1	tps187650, tho142780, CC00G90420	001015
Sig3	Armbrust (1999)	ND	Sm_t00014525-RA Sm_t00012303-RA	3.03E-10 1.15E-10	MMETSP0321-DN11213_c2_g1_i1.p1	tps23700, CC00G26700	001015
DNAH9/11/17	Ferrante et al. (2019)	MTRINITY_DN17388_c0_g1_i3	Sm_t00001714-20-RA*	All significant*	Missing	tps140350, CC00G148780	000072
DNAI2	Ferrante et al. (2019)	STRINITY_DN18690_c0_g1_i1	Sm_t00001899-RA	0.35	MMETSP0321-DN10800_c3_g1_i1.p1	tho18620, tho481440, CC00G41890, tps143600	004104
DNAH5/8	Ferrante et al. (2019)	Pc32863_g3_i1	Sm_t00013147-RA	0.0072	MMETSP0321-DN11382_c3_g2_i1.p1	tho15560, tps104840, CC00G202730	000072
TCTEX1D	Ferrante et al. (2019)	MTRINITY_DN20279_c0_g1_i1	Sm_t00007855-RA	Filtered	MMETSP0322-DN9821_c0_g1_i1.p1	CC00G59130, tho148540, tps73620	003143
DYNLRB	Ferrante et al. (2019)	STRINITY_DN2071_c0_g1_i1	Sm_t00019223-RA	Filtered	MMETSP0321-DN13408_c0_g1_i1.p1	CC00G13880	001545

Tctex1-like	Ferrante et al. (2019)	STRINITY_DN18525_c0_g1_i1	Sm_t00014345-RA	Filtered	MMETSP0321-DN4818_c0_g2_i1.p2	CC00G13760, tho460520	002302
DRC4	Ferrante et al. (2019)	STRINITY_DN15037_c0_g1_i1	Sm_t00009436-RA Sm_t00020708-RA	5.07E-10 Filtered	MMETSP1362-DN5944_c0_g2_i1.p1	CC00G152160, tho321800, tps110660	023378
DRC2	Ferrante et al. (2019)	STRINITY_DN9819_c0_g1_i1	*Missing*	*missing*	MMETSP0321-DN4715_c0_g1_i1	tho224980, tps203950, CC00G172940	005821
Tubulin beta chain	Ferrante et al. (2019)	STRINITY_DN3540_c0_g1_i1	*Missing*	*missing*	MMETSP1362-DN7090_c0_g1_i1	tps206490, CC00G11890	000065

Table S6: *List of 119 vegetative and 8 sexual RNA-seq libraries from the S. robusta expression atlas used to verify specificity of markers for sex.*

Experiment	Treatment	# Samples	Source
Bacterial AHL	Control	3	Stock et al. (2020)
Bacterial AHL	C14	3	Stock et al. (2020)
Bacterial AHL	OXO	3	Stock et al. (2020)
Bacterial AHL	TA14	2	Stock et al. (2020)
Bacterial exudates	Control	3	Cirri et al. (2019)
Bacterial exudates	Maribacter	3	Cirri et al. (2019)
Bacterial exudates	Roseovarius	3	Cirri et al. (2019)
Decadienal	Control	3	Osuna-Cruz et al. (2020)
Decadienal	Decadienal	3	Osuna-Cruz et al. (2020)
Diurnal cycle	2h	3	Bilcke et al. (2021)
Diurnal cycle	6h	3	Bilcke et al. (2021)
Diurnal cycle	10h	3	Bilcke et al. (2021)
Diurnal cycle	14h	3	Bilcke et al. (2021)
Diurnal cycle	18h	3	Bilcke et al. (2021)
Diurnal cycle	22h	3	Bilcke et al. (2021)
Diurnal cycle	26h	3	Bilcke et al. (2021)
Diurnal cycle	30h	3	Bilcke et al. (2021)
Diurnal cycle	34h	3	Bilcke et al. (2021)
Diurnal cycle	38h	3	Bilcke et al. (2021)
Diurnal cycle	42h	3	Bilcke et al. (2021)
Diurnal cycle	46h	3	Bilcke et al. (2021)
H2O2 and Salt	Control	3	Osuna-Cruz et al. (2020)
H2O2 and Salt	H2O2	3	Osuna-Cruz et al. (2020)
H2O2 and Salt	High salinity	3	Osuna-Cruz et al. (2020)

H2O2 and Salt	Low salinity	3	Osuna-Cruz et al. (2020)
High light	Control	3	Osuna-Cruz et al. (2020)
High light	30 min	3	Osuna-Cruz et al. (2020)
High light	6h	3	Osuna-Cruz et al. (2020)
N & P depletion	Control (48h)	2	Osuna-Cruz et al. (2020)
N & P depletion	Control (72h)	2	Osuna-Cruz et al. (2020)
N & P depletion	N depleted (48h)	2	Osuna-Cruz et al. (2020)
N & P depletion	N depleted (72h)	2	Osuna-Cruz et al. (2020)
N & P depletion	P depleted (48h)	2	Osuna-Cruz et al. (2020)
N & P depletion	P depleted (72h)	2	Osuna-Cruz et al. (2020)
Sexual reproduction	Control P	3	Osuna-Cruz et al. (2020)
Sexual reproduction	Control GZ	3	Osuna-Cruz et al. (2020)
Sexual reproduction	Control A	3	Osuna-Cruz et al. (2020)
Silica	Control	3	Osuna-Cruz et al. (2020)
Silica	Depleted	3	Osuna-Cruz et al. (2020)
Temperature	Control	3	Osuna-Cruz et al. (2020)
Temperature	30°C	3	Osuna-Cruz et al. (2020)
Temperature	4°C	3	Osuna-Cruz et al. (2020)

Table S7: Gene family name, taxonomical distribution and gene identifiers of four selected conserved marker gene families. *avLFC: the average log2 fold change during sexual reproduction for all significantly differentially expressed time points across the gene family.* *Two adjacent partial gene models separately detected by the data-driven marker analysis, later manually curated by merging.

**Name**	**PLAZA Diatoms HOM02SEM GF**	***S. marinoi***	***P. multistriata***	***S. robusta***	***C. closterium***	**avLFC**
M1	020815	NA	Pmu0035440	Sro453_g146170	Ccl_11047	8.73
M2	010892	NA	Pmu0059350	Sro810_g205790	Ccl_13876	5.96
M3	008092	Sm_t00009448-RA<br>Sm_t00020721-RA*<br>Sm_t00020722-RA *	Pmu0010180	Sro587_g171310	Ccl_13049	5.69
M4	015574	NA	Pmu0108890	Sro37_g023460<br>Sro37_g023470	Ccl_2381	5.38

Table S8: Expression statistics and thresholds for different sex marker families, based on their expression in non-sexual vegetative conditions. *Values in transcripts per million (TPM).*

Sex marker	Mean	95% percentile	Coefficient of Variation
M1	0.14	0.77	3.20
M2	1.06	9.36	2.90
M3	3.15	29.40	3.93
M4	1.32	5.66	2.54
SIG1	11.54	28.20	0.98
DNAH5/8	0.56	1.37	0.88
DNAH9/11/17	0.35	0.68	0.79
DRC4	0.09	0.40	1.96

Table S9: Expression statistics and thresholds for different control marker families, based on their expression in all RNA-seq conditions. *Values in transcripts per million (TPM).*

Control marker	Mean	95% percentile	Coefficient of Variation
C1: HOM02SEM012764	0.60	1.87	5.59
C2: HOM02SEM013929	2.45	10.29	0.87
C3: HOM02SEM017748	3.56	7.50	2.09
C4: HOM02SEM010612	2.66	5.02	0.76

Table S10: Number of MAGs retrieved for different diatom taxonomic assignments. *The table shows the total number of diatom MAGs at the highest taxonomic resolution available, as well as the subset of those MAGs that exhibit sexual signal.*

**Genus**	**Group**	**Number of MAGs**	**Number of MAGs exhibiting sexual signal**
Attheya	Centric	1	0
Chaetoceros	Centric	11	5
Leptocylindrus	Centric	1	1
Minidiscus	Centric	2	2
Minutocellus	Centric	2	0
Odontella	Centric	1	1
Skeletonema	Centric	3	3
Thalassiosira	Centric	5	2
Unknown Coscinodiscophyceae	Centric	1	0
Unknown Cymatosiraceae	Centric	1	0
Unknown Mediophyceae	Centric	1	0
Cylindrotheca	Pennate	2	2
Fragilariopsis	Pennate	5	4
New Bacillariaceae	Pennate	4	0
Pseudo-nitzschia	Pennate	7	4
Synedropsis	Pennate	1	0
Unknown Bacillariaceae	Pennate	1	0
Unknown Bacillariophyceae	Pennate	1	0
Unknown Bacillariophyta	Pennate	2	0

Table S11: Number of genes retrieved after each step of the gene identification workflow in Tara Oceans MAGs. *The table includes the number of genes retrieved after the initial hmmsearch, followed by filtering based on bit score and e-value, taxonomy-based selection for diatom hits, and finally, phylogenetic selection to refine the gene set. Each column represents a stage in the filtering process.*

Marker	Raw hits	High-quality raw hits	Diatom high quality hits	Diatom hits after phylogenetic selection
**M1**	1159	186	30	7
**M2**	576	150	31	20
**M3**	330	114	111	35
**M4**	61	22	21	16
**SPO11-2**	542	444	86	42
**DNAH5/8**	16872	14553	246	55
**DNAH9/11/17**	17155	14623	251	49
**DRC4**	419	330	23	21
**SIG1**	4045	1497	79	19

Table S12. Summary of retrieved data based on selection criteria involving the co-expression of the gene SPO11-2 with additional markers for centric and pennate diatoms. *The table details the number of stations, genera, and metagenome-assembled genomes (MAGs) identified for different combinations of marker genes. For centric diatoms (top section), the selection involved a broader set of markers (SPO11-2, DNAH5/8, DNAH9/11/17, SIG1, M3, DRC4), and for pennate diatoms (bottom section), a different marker set (SPO11-2, M1, M2, M3, M4) was used. Both SPO11-2 and the sexual markers are considered expressed if they surpass the defined TPM thresholds and have at least two reads. The analysis is split into these two diatom groups to reflect their different genetic markers.*

**Centric Diatoms** (Marker List: SPO11-2, DNAH5/8, DNAH9/11/17, SIG1, M3, DRC4)			
**Filtering Criterion**	**Number of stations**	**Numbers of genera**	**Number of MAGs**
SPO11-2 + at least 1 marker	42	6	14
SPO11-2 + at least 2 markers	38	6	13
SPO11-2 + at least 3 markers	25	6	11
SPO11-2 + at least 4 markers	8	5	8
SPO11-2 + 5 markers	4	2	2
**Pennate Diatoms** (Marker List: SPO11-2, M1, M2, M3, M4)			
**Filtering Criterion**	**Number of stations**	**Numbers of genera**	**Number of MAGs**
SPO11-2 + at least 1 marker	50	3	10
SPO11-2 + at least 2 markers	24	3	8
SPO11-2 + at least 3 markers	10	2	5
SPO11-2 + 4 markers	2	1	1

Table S13: Assembly metrics and taxonomic annotation for Tara Oceans diatom MAGs*. This table shows the taxonomic classification of each Metagenome-Assembled Genome (MAG), along with whether it exhibits a sexual reproduction signal (*Sex MAG *= "yes" or "no") and the values of various assembly metrics (e.g.,* total_length*,* N50*,* ANVIO_completion*,* ANVIO_redundancy*). These data were used to compare the assembly characteristics of MAGs showing sexual signals to those without, as presented in the supplementary figure S30. Total length: sum of all contig lengths in the MAG. N50: the contig length at which half of the total assembly length is contained in contigs of that length or longer. ANVIO completion and ANVIO redundancy: estimated completeness and redundancy from the Anvi'o pipeline.*

**TARA_MAG_ID**	**Genus**	**Total length**	**Num contigs**	**N50**	**ANVIO completion**	**ANVIO redundancy**	**Sex MAG**
TARA_AON_82_MAG_00338	*Minutocellus*	18254396	4021	4676	56.63	8.43	No
TARA_AOS_82_MAG_00050	Unknown_Cymatosiraceae	25177467	1952	18760	87.95	3.61	No
TARA_AOS_82_MAG_00147	*Thalassiosira*	15619125	4691	3214	20.48	6.02	No
TARA_ARC_108_MAG_00137	New_Bacillariaceae_02	40678489	7917	5534	83.13	4.82	No
TARA_ARC_108_MAG_00165	*Synedropsis*	39329959	7674	5575	83.13	9.64	No
TARA_ARC_108_MAG_00209	*Minutocellus*	27486254	4714	6649	75.9	8.43	No
TARA_ARC_108_MAG_00212	*Pseudo-nitzschia*	28586333	5125	6113	73.49	7.23	No
TARA_ARC_108_MAG_00222	Unknown_Bacillariophyta	40136874	7131	6371	48.19	2.41	No
TARA_ARC_108_MAG_00230	*Pseudo-nitzschia*	20673871	4737	4490	50.6	4.82	No
TARA_ARC_108_MAG_00232	*Chaetoceros*	10655193	2914	3561	19.28	3.61	No
TARA_ARC_108_MAG_00238	Unknown_Bacillariophyta	13995552	947	18194	40.96	1.2	No
TARA_ARC_108_MAG_00239	*Attheya*	11711613	3186	3605	42.17	10.84	No
TARA_ARC_108_MAG_00267	*Chaetoceros*	29563790	7475	3927	53.01	6.02	no
TARA_ARC_108_MAG_00269	*Thalassiosira*	12245782	2907	4204	6.02	0	no
TARA_ARC_108_MAG_00276	New_Bacillariaceae_02	27285525	6391	4310	49.4	3.61	no
TARA_ARC_108_MAG_00286	Unknown_Bacillariaceae	13435158	3584	3662	2.41	0	no
TARA_ARC_108_MAG_00313	*Thalassiosira*	1.3E+08	33811	3761	36.14	7.23	no
TARA_IOS_50_MAG_00125	New_Bacillariaceae_01	12823134	3456	3634	45.78	2.41	no
TARA_MED_95_MAG_00399	*Chaetoceros*	17395980	4032	4435	53.01	2.41	no
TARA_MED_95_MAG_00424	Unknown_Bacillariophyceae	25165269	3794	7882	75.9	6.02	no
TARA_MED_95_MAG_00467	Unknown_Coscinodiscophyceae	58956988	13079	4707	75.9	13.25	no
TARA_PSE_93_MAG_00171	*Chaetoceros*	36009581	4704	9415	83.13	10.84	no
TARA_PSE_93_MAG_00213	*Pseudo-nitzschia*	17103317	3743	4685	46.99	1.2	no
TARA_PSW_86_MAG_00256	*Chaetoceros*	44361495	9477	4855	68.67	3.61	no
TARA_PSW_86_MAG_00281	New_Bacillariaceae_01	17426785	4498	3824	49.4	7.23	no
TARA_SOC_28_MAG_00060	*Chaetoceros*	12202481	3483	3404	25.3	3.61	no
TARA_SOC_28_MAG_00071	*Fragilariopsis*	16160340	3998	3995	46.99	1.2	no
TARA_SOC_28_MAG_00077	Unknown_Mediophyceae	93608600	18094	5648	71.08	9.64	no
TARA_AON_82_MAG_00159	*Cylindrotheca*	24267360	2892	10926	87.95	7.23	yes
TARA_AOS_82_MAG_00176	*Fragilariopsis*	28681957	6363	4642	62.65	1.2	yes
TARA_ARC_108_MAG_00108	*Leptocylindrus*	40473145	7090	6501	91.57	9.64	yes
TARA_ARC_108_MAG_00116	*Chaetoceros*	32722577	3326	13011	91.57	10.84	yes
TARA_ARC_108_MAG_00122	*Fragilariopsis*	35575995	4966	8878	85.54	6.02	yes

TARA_ARC_108_MAG_00138	*Skeletonema*	29129855	3476	10535	93.98	15.66	yes
TARA_ARC_108_MAG_00187	*Fragilariopsis*	41050103	8306	5215	71.08	0	yes
TARA_ARC_108_MAG_00189	*Chaetoceros*	34156899	4441	10176	81.93	10.84	yes
TARA_ARC_108_MAG_00217	*Chaetoceros*	33988865	6182	6060	74.7	10.84	yes
TARA_ARC_108_MAG_00219	*Minidiscus*	33514166	6685	5359	71.08	10.84	yes
TARA_ARC_108_MAG_00228	*Pseudo-nitzschia*	18046799	4801	3743	37.35	2.41	yes
TARA_ARC_108_MAG_00252	*Thalassiosira*	10800609	3219	3239	9.64	1.2	yes
TARA_ARC_108_MAG_00253	*Skeletonema*	23295314	5001	4822	54.22	8.43	yes
TARA_IOS_50_MAG_00056	*Cylindrotheca*	25608881	1568	25257	93.98	7.23	yes
TARA_IOS_50_MAG_00115	*Pseudo-nitzschia*	22647878	3259	8502	69.88	1.21	yes
TARA_MED_95_MAG_00394	*Skeletonema*	23791115	4390	5905	72.29	12.05	yes
TARA_PSE_93_MAG_00253	*Chaetoceros*	24577184	5729	4407	56.63	4.82	yes
TARA_PSW_86_MAG_00222	*Pseudo-nitzschia*	22525297	2997	9314	77.11	4.82	yes
TARA_PSW_86_MAG_00236	*Pseudo-nitzschia*	32295870	6423	5278	75.9	6.02	yes
TARA_PSW_86_MAG_00261	*Thalassiosira*	32906914	6352	5589	79.52	22.89	yes
TARA_PSW_86_MAG_00276	*Minidiscus*	24492844	6619	3626	46.99	7.23	yes
TARA_SOC_28_MAG_00031	*Fragilariopsis*	38589172	7206	5859	79.52	1.20	yes
TARA_SOC_28_MAG_00037	*Chaetoceros*	30419621	3201	13607	85.54	10.84	yes
TARA_SOC_28_MAG_00049	*Odontella*	1.32E+09	419520	3445	75.91	6.02	yes

Supplementary Notes

Note S1: Proof-of-concept for marker identification using RT-qPCR

We performed a reverse transcriptase quantitative PCR (RT-qPCR) experiment on sexualized and vegetative cultures of the pennate *Cylindrotheca closterium* in order to assess the feasibility of picking up co-expressed marker genes using this method. Specifically, *C. closterium* strains of opposite mating type, A6 (DCG 0980, MT-) and MC4 (DCG 1267, MT+), were obtained from the Belgian Coordinated Collection of Microorganisms (BCCM/DCG, https://bccm.belspo.be/about-DCG). Cells were maintained in Guillard's F/2 Marine Water enrichment solution (Sigma-Aldrich) supplemented with silica (30 mg/L) at 18 °C, a light intensity of 20 µmol photons m^{-2} s^{-1} and a 12:12h L:D cycle, and dark arrested for 24 hours before the experiment. To induce sexual reproduction, strains A6 and MC4 were mixed at equal densities (40,000 cells/mL per strain) and afterwards kept in continuous light at 18 °C and a light intensity of 20 µmol photons m^{-2} s^{-1}. Crossings were harvested 15 hours after induction of sexual reproduction by vacuum filtration on Versapor filters (3 µm pore size, 25 mm diameter, Pall Corporation, USA). At the time of harvesting, unpaired parental cells, paired cells, gametes and zygotes were present in the sexual cultures. As a control, vegetative A6 and MC4 cultures were incubated under the same conditions as the crossings, and harvested after 15 hours with equal densities of each strain on the filter (40,000 cells/mL per strain). All cultures were flash frozen in liquid nitrogen immediately after harvesting and stored at -80 °C.

RNA was extracted with the RNeasy Plant Mini Kit (Qiagen, The Netherlands) and an additional DNase treatment was performed with the RNase-Free Dnase Set (Qiagen, The Netherlands). Purity of the RNA was checked by spectrophotometry (Nanodrop, ThermoFisher Scientific, USA), and RNA concentration and integrity were determined by a 5200 Fragment Analyzer System (Agilent Technologies, USA). cDNA synthesis was performed according to the iScript™ cDNA Synthesis Kit (Bio-Rad, USA). Primers for four sex marker genes and reference genes were designed with Primer3Plus [2] using qPCR settings (**Table S3**). Sex marker genes used were Ccl_11047 (Marker 1), Ccl_13876 (Marker 2), Ccl_13049 (Marker 3) and Ccl_2381 (Marker 4). Reference genes used were Ccl_2393 (histone 2B; H2B), Ccl_21857 (TATA box binding protein; TATA) and Ccl_5938 (β-tubulin; TubB2).

RT-qPCR was conducted on a LightCycler® 480 Instrument II using the LightCycler® 480 SYBR Green I Master kit (Roche, Germany) supplemented with custom primers. The qPCR mixture consisted of 1 µL cDNA (24 ng/µL), 0.5 µL of forward and reverse primer (10 µM), 2.5 µL SYBR Green Master Mix and 0.5 µL DNase & RNase free water in a total volume of 5 µL. The PCR program comprised of a pre-incubation step of 5 mins at 95 °C and an amplification step of 45 cycles consisting of 10s at 95 °C, 20 s at 60 °C and 20s at 72 °C. A melting curve was generated by heating for 5 s at 95 °C, 1 min at 65 °C and then continuously at 97 °C. After the generation of the melting curve, the PCR products were cooled down to 40 °C for 10 s. Ct values were obtained by running the “Abs Quant/2nd Derivative Max” analysis and melting curves were obtained by running “Tm calling/Melting curve analysis” of the LightCycler® 480 Software.

Relative gene expression in terms of relative fold changes (RFCs) were calculated using the delta-delta Ct ($2^{-\Delta\Delta Ct}$) method. For statistical analysis, the RFCs were log2 transformed with an offset of 0.01 to account for fold changes of zero. The Shapiro-Wilk test and F-test were used to check for normality and homogeneity of variances respectively. Statistical significance between RFCs in the sexual and control samples was calculated using a Two Sample t-test.

For all markers, the relative fold change was significantly higher in the sexual compared to the control cultures (Two Sample t-test, $p < 0.05$, **Figure S5**). The RFCs of the sexual cultures of marker 1 and 4 were markedly higher than the RFCs of marker 2 and 3, suggesting that marker 1 and 4 might be more sensitive for picking up early stages of sexual reproduction in natural samples. As auxospores were not present in the sexual cultures yet, future RT-qPCR experiments could focus on the feasibility of the markers to detect later stages of sexual reproduction. Given that some background signal was picked up by RT-qPCR in vegetative conditions, we recommend to assess the co-expression of the four markers to make inferences about sex in natural samples rather than using a single marker to avoid false positive signals. Altogether, this experiment shows the potential of using RT-qPCR with sex marker genes to detect sexual reproduction in natural diatom populations, which will be essential in understanding the phenology of diatom sexual reproduction and population dynamics.

Note S2: Generation and characterization of microcosm *de novo* transcriptome

Based on the >600M paired-end Illumina reads of the microcosm experiment, a Trinity *de novo* transcriptome was assembled containing 868,920 individual transcripts. Taxonomic binning recovered transcripts belonging to diatoms (40%), as well as green algae and plants (8%), animals (2%), fungi (5%) and bacteria (26%) (**Figures S15, S16**). Hence, we captured a diverse community of uni- and multicellular species from various taxa, allowing us to study the specificity of our sex marker approach. Notably, taxonomic binning of the raw reads revealed a clear shift in abundance of diatoms without (57%) and with (77%) a salinity shock treatment, suggesting a higher mortality of non-diatoms and a higher salt-tolerance of diatom species (**Figures S15, S16**). After read mapping (**Figure S17A**), multidimensional scaling of samples by their leading log fold changes showed clear clustering by salinity treatment, and to a lesser extent by time (24h, 48h after treatment) (**Figure S17B**).

Note S3: Methodological Considerations on *Tara* metabarcoding

For several genera, we observed marked differences between OTU abundances depending on whether they are derived from the V4 or V9 regions of the 18S rDNA. Primer specificity and inherent genetic variability could have contributed to the observed distinct patterns, emphasizing the importance of understanding characteristics in metabarcoding studies on diatoms and plankton in general [3–5].

Note S4: Biogeography of sexual reproduction events in the *Tara* Oceans dataset

The co-expression analysis revealed sexual reproduction in 9 diatom genera across 54 locations globally (**Figure 5a**). The majority of genera exhibited distinct patterns, with *Chaetoceros*, *Fragilariopsis*, *Leptocylindrus*, *Odontella*, and *Skeletonema* showing a preference for cold waters. Sexually active *Chaetoceros* species dominated the Arctic Ocean and were also present in the Southern Ocean, near the Gibraltar Strait, and in the South Pacific Ocean (station 092 - a location characterized by the cold waters of the Chile upwelling system). One *Chaetoceros* Metagenome-Assembled Genomes (MAG) co-expressed sexual markers in Arctic stations 163 and 188, with *Chaetoceros* cells expressing markers in both picoplankton and nanoplankton size groups (**Figure 5b**). IFCB observations confirmed the presence of both *Chaetoceros* vegetative cells and spermatocytes in these stations, with cell diameters consistent with the detected expression signal. Additionally, a possible *Chaetoceros* egg cell was identified in station 188. The latter also hosted signals of sexual reproduction by *Skeletonema* (**Figure 5a**). *Fragilariopsis* MAGs displayed a cold-water sexual reproduction in the Arctic and Southern Ocean, while *Leptocylindrus* exhibited a cold-water pattern in the South Atlantic Ocean, in the Pacific-influenced side of the Arctic Ocean, and in the Labrador Sea, here accompanied by the identification of both vegetative *Leptocylindrus* cells and a sexual spore (**Figure 5b**). *Odontella* displayed sexual reproduction only in the Southern Ocean, while *Skeletonema* exhibited activity across the whole Arctic Ocean, and near the Gibraltar Strait. *Cylindrotheca*, with a non-polar pattern, showed sexual markers co-expression across the Indian Ocean, the Northern Atlantic Ocean, the Mediterranean Sea, and at one Pacific Ocean location. *Minidiscus*, *Pseudo-nitzschia* and *Thalassiosira* exhibited a widespread occurrence of sexual activity, encompassing the Mediterranean Sea, the Indian Ocean, both the Southern and Northern Atlantic Ocean, the Arctic Ocean, and numerous locations across the tropical Pacific Ocean (**Figure 5a**). Importantly, the IFCB images never contained sexual cell types in stations where the corresponding genus was not expressing any of the marker genes.

Note S5: influence of depth on sexual reproduction

We observed that sexual reproduction occurs predominantly at the ocean surface, in accordance with field observations, where reproductive stages of *Pseudo-nitzschia* and *Aulacoseira* predominantly occurred in surface layers [8–10]. However, more fine-grained profiling of marker expression will be needed to assess the proposed effects of depth and cell sinking on the induction of sex. Some diatoms actively modulate their position in the water column through buoyancy control [11]; moreover, sexual reproduction gives rise to sedimentation of empty frustules which contributes to the vertical silica flux towards the sediment [12]. Hence, monitoring of sexuality events is a key element in our understanding of global biogeochemical cycles, particularly those involving silicon.

Bibliography

1. Wu, T. *et al.* clusterProfiler 4.0: A universal enrichment tool for interpreting omics data. *Innovation (Camb)* **2**, 100141 (2021).

2. Untergasser, A. *et al.* Primer3Plus, an enhanced web interface to Primer3. *Nucleic Acids Res* **35**, W71-74 (2007).

3. Nanjappa, D., Kooistra, W. H. C. F. & Zingone, A. A reappraisal of the genus *Leptocylindrus* (Bacillariophyta), with the addition of three species and the erection of *Tenuicylindrus* gen. nov. *J Phycol* **49**, 917–936 (2013).

4. Piredda, R. *et al.* Diversity and temporal patterns of planktonic protist assemblages at a Mediterranean Long Term Ecological Research site. *FEMS Microbiol Ecol* **93**, fiw200 (2017).

5. Tragin, M., Zingone, A. & Vaulot, D. Comparison of coastal phytoplankton composition estimated from the V4 and V9 regions of the 18S rRNA gene with a focus on photosynthetic groups and especially Chlorophyta. *Environ Microbiol* **20**, 506–520 (2018).

6. Annunziata, R. *et al.* Trade-off between sex and growth in diatoms: Molecular mechanisms and demographic implications. *Science Advances* **8**, eabj9466 (2022).

7. Basu, S. *et al.* Finding a partner in the ocean: molecular and evolutionary bases of the response to sexual cues in a planktonic diatom. *New Phytologist* **215**, 140–156 (2017).

8. Jewson, D. H. & Granin, N. G. Cyclical size change and population dynamics of a planktonic diatom, *Aulacoseira baicalensis*, in Lake Baikal. *European Journal of Phycology* **50**, 1–19 (2015).

9. Holtermann, K. E., Bates, S. S., Trainer, V. L., Odell, A. & Armbrust, E. V. Mass sexual reproduction in the toxigenic diatoms *Pseudo-nitzschia* australis and *P. pungens* (Bacillariophyceae) on the Washington coast, USA. *Journal of Phycology* **46**, 41–52 (2010).

10. Sarno, D., Zingone, A. & Montresor, M. A massive and simultaneous sex event of two *Pseudo-nitzschia* species. *Deep-Sea Research Part II: Topical Studies in Oceanography* **57**, 248–255 (2010).

11. Du Clos, K. T., Karp-Boss, L., Villareal, T. A. & Gemmell, B. J. *Coscinodiscus wailesii* mutes unsteady sinking in dark conditions. *Biology Letters* **15**, 20180816 (2019).

12. Crawford, R. M. The role of sex in the sedimentation of a marine diatom bloom. *Limnology and Oceanography* **40**, 200–204 (1995).
