# Supplementary material for: Conserved genetic markers reveal widespread diatom sexual reproduction in the global ocean
Source: Nat Commun. 2025 Nov 14;16:10029. doi: 10.1038/s41467-025-65296-9 (PMC12618581; doi:10.1038/s41467-025-65296-9)
Supplement: Supplementary file 2 — Reporting Summary [file 41467_2025_65296_MOESM2_ESM.pdf]

nature portfolio

Corresponding author(s): Maria Immacolata Ferrante
Klaas Vandepoele
Wim Vyverman

Last updated by author(s): 11/08/2025

# Reporting Summary

Nature Portfolio wishes to improve the reproducibility of the work that we publish. This form provides structure for consistency and transparency in reporting. For further information on Nature Portfolio policies, see our Editorial Policies and the Editorial Policy Checklist.

## Statistics

For all statistical analyses, confirm that the following items are present in the figure legend, table legend, main text, or Methods section.

| n/a | Confirmed | |
|---|---|---|
| ☐ | ☒ | The exact sample size (*n*) for each experimental group/condition, given as a discrete number and unit of measurement |
| ☐ | ☒ | A statement on whether measurements were taken from distinct samples or whether the same sample was measured repeatedly |
| ☐ | ☒ | The statistical test(s) used AND whether they are one- or two-sided<br>*Only common tests should be described solely by name; describe more complex techniques in the Methods section.* |
| ☐ | ☒ | A description of all covariates tested |
| ☐ | ☒ | A description of any assumptions or corrections, such as tests of normality and adjustment for multiple comparisons |
| ☐ | ☒ | A full description of the statistical parameters including central tendency (e.g. means) or other basic estimates (e.g. regression coefficient) AND variation (e.g. standard deviation) or associated estimates of uncertainty (e.g. confidence intervals) |
| ☒ | ☐ | For null hypothesis testing, the test statistic (e.g. *F*, *t*, *r*) with confidence intervals, effect sizes, degrees of freedom and *P* value noted<br>*Give P values as exact values whenever suitable.* |
| ☒ | ☐ | For Bayesian analysis, information on the choice of priors and Markov chain Monte Carlo settings |
| ☒ | ☐ | For hierarchical and complex designs, identification of the appropriate level for tests and full reporting of outcomes |
| ☐ | ☒ | Estimates of effect sizes (e.g. Cohen's *d*, Pearson's *r*), indicating how they were calculated |

*Our web collection on statistics for biologists contains articles on many of the points above.*

## Software and code

Policy information about availability of computer code

| | |
|---|---|
| Data collection | No software was used for data collection |
| Data analysis | Transdevcoder v5.0.2<br>Diamond v2.0.14<br>FastQC v0.11.2<br>MultiQC v1.7<br>Trimmomatic v0.36<br>Salmon v1.3.0/v1.8.0<br>EdgeR 3.30.3<br>MEGAX 11.0.8<br>HMMer 3.1b2<br>MAFFT v7.453/v7.490<br>DADA2 1.14.1<br>PhyloSeq 1.36.0<br>IQ-tree v2.1.3<br>BBMap v38.98<br>rnaSPAdes v3.14.1<br>Transdecoder v5.0.2<br>Diamond v2.0.14 |

Kraken2 v2.1.2
TrimAl v1.4.1
FigTree v1.4.3

For manuscripts utilizing custom algorithms or software that are central to the research but not yet described in published literature, software must be made available to editors and reviewers. We strongly encourage code deposition in a community repository (e.g. GitHub). See the Nature Portfolio guidelines for submitting code & software for further information.

## Data

Policy information about availability of data

All manuscripts must include a data availability statement. This statement should provide the following information, where applicable:

- Accession codes, unique identifiers, or web links for publicly available datasets
- A description of any restrictions on data availability
- For clinical datasets or third party data, please ensure that the statement adheres to our policy

Raw paired-end Illumina reads of the Scheldt microcosm experiment are available as a European Nucleotide Archive study PRJEB61014, with Sample accessions ERS14855523-ERS14855528 (metatranscriptomics) and ERS16476494-ERS16476502 (metabarcoding).

All other relevant data have been deposited to Zenodo: https://zenodo.org/records/11258439, including including (1) profile HMMs for all markers, (2) reference proteins for each marker for phylogenetic selection, (3) manually curated gene models, (4) differential expression and gene family tables for comparative transcriptomics, (5) transcripts and differential expression tables of the Scheldt metatranscriptome (both relative to the entire co-assembly and normalized per Thalassiosirales clade), (6) expression levels (# reads, transcripts per million) of genes encoded on diatom MAGs that express SPO11-2 in the Tara Oceans dataset., (7) phylogenetic trees of marker proteins and their metatranscriptome homologs. Selected clades containing orthologous markers are indicated (8) maps with co-expression on the MAG level in the Tara Oceans dataset, and (9) a table with observations of sex in the public IFCB data from EcoTaxa

## Research involving human participants, their data, or biological material

Policy information about studies with human participants or human data. See also policy information about sex, gender (identity/presentation), and sexual orientation and race, ethnicity and racism.

| | |
|---|---|
| Reporting on sex and gender | *Use the terms sex (biological attribute) and gender (shaped by social and cultural circumstances) carefully in order to avoid confusing both terms. Indicate if findings apply to only one sex or gender; describe whether sex and gender were considered in study design; whether sex and/or gender was determined based on self-reporting or assigned and methods used. Provide in the source data disaggregated sex and gender data, where this information has been collected, and if consent has been obtained for sharing of individual-level data; provide overall numbers in this Reporting Summary. Please state if this information has not been collected. Report sex- and gender-based analyses where performed, justify reasons for lack of sex- and gender-based analysis.* |
| Reporting on race, ethnicity, or other socially relevant groupings | *Please specify the socially constructed or socially relevant categorization variable(s) used in your manuscript and explain why they were used. Please note that such variables should not be used as proxies for other socially constructed/relevant variables (for example, race or ethnicity should not be used as a proxy for socioeconomic status). Provide clear definitions of the relevant terms used, how they were provided (by the participants/respondents, the researchers, or third parties), and the method(s) used to classify people into the different categories (e.g. self-report, census or administrative data, social media data, etc.) Please provide details about how you controlled for confounding variables in your analyses.* |
| Population characteristics | *Describe the covariate-relevant population characteristics of the human research participants (e.g. age, genotypic information, past and current diagnosis and treatment categories). If you filled out the behavioural & social sciences study design questions and have nothing to add here, write "See above."* |
| Recruitment | *Describe how participants were recruited. Outline any potential self-selection bias or other biases that may be present and how these are likely to impact results.* |
| Ethics oversight | *Identify the organization(s) that approved the study protocol.* |

Note that full information on the approval of the study protocol must also be provided in the manuscript.

# Field-specific reporting

Please select the one below that is the best fit for your research. If you are not sure, read the appropriate sections before making your selection.

☐ Life sciences ☐ Behavioural & social sciences ☒ Ecological, evolutionary & environmental sciences

For a reference copy of the document with all sections, see nature.com/documents/nr-reporting-summary-flat.pdf

# Ecological, evolutionary & environmental sciences study design

All studies must disclose on these points even when the disclosure is negative.

| | |
|---|---|
| Study description | [1] Integration of sexual reproduction RNA-sequencing time series datasets from four diatom species |

| | |
|---|---|
| | [2] Metabarcoding and metatranscriptome study of a microcosm derived from a natural community from the freshwater Scheldt estuary<br>[3] Bio-informatic analyses on publicly available metagenomic, metatranscriptomic, metabarcoding and automated imaging resources associated with the Tara Oceans expedition |
| Research sample | [1] Sexually induced cultures of four different diatom species: Seminavis robusta, Cylindrotheca closterium, Pseudo-nitzschia multistriata and Skeletonema marinoi.<br>[2] A natural phytoplankton sample from the freshwater zone of the tidal Scheldt estuary near the city of Ghent (51°00'15.6"N, 3°48'19.3"E)<br>[3] Metagenomic, metatranscriptomic, metabarcoding and imaging flowCytoBot data have been collected during the Tara Oceans expeditions (Sunagawa et al. 2020, Delmont et al. 2022) |
| Sampling strategy | [1] All experiments were previously carried out by our research groups. Sampling strategies were reported in the targeted studies of each species (Ferrante et al. 2019, Osuna-Cruz et al. 2020, Bilcke et al. 2021, Annunziata et al. 2022, Audoor et al. 2024)<br>[2] While metatranscriptome experiments often do not involve replication, we did harvest replicate samples during the microcosm experiment (four independent replicates of salt treated, and two replicates of control cultures, spread over two time points) allowing us to perform differential expression analysis while maintaining a high sequencing depth (> 100M paired-end reads per sample) |
| Data collection | [1] Data collection was previously described in Ferrante et al. 2019, Osuna-Cruz et al. 2020, Bilcke et al. 2021, Annunziata et al. 2022, Audoor et al. 2024<br>[2] Natural samples were collected by Luz Amadei Martínez through filtering on a plankton net with a mesh size of 10 µm, followed by distribution over large 150-mL culture flasks. |
| Timing and spatial scale | [2] Natural phytoplankton samples were collected on 20/05/2022 from the freshwater zone of the tidal Scheldt estuary near the city of Ghent (51°00'15.6"N, 3°48'19.3"E) |
| Data exclusions | None of the collected samples was excluded from analysis |
| Reproducibility | Where possible, experimental results were validated using complimentary data types. The markers for sex were validated using RT-qPCR (culture conditions) and the microcosm metatranscriptome experiment (natural conditions), which was in turn validated using microscopy and metabarcoding. The identification of sexual hotspots for some Tara Arctic stations could be confirmed using imaging flow cytometry data from the same expedition. |
| Randomization | Scheldt microcosm flasks were positioned in a randomized way during incubation. All other steps were performed in a single time/run to avoid batch effects. |
| Blinding | NA |

Did the study involve field work? ☒ Yes ☐ No

## Field work, collection and transport

| | |
|---|---|
| Field conditions | Ongoing seasonal diatom bloom (chlorophyll a ~50 µg/L) in the freshwater (~0.4 ppt) tidal reaches of the Scheldt estuary. |
| Location | 51°00'15.6"N, 3°48'19.3"E |
| Access & import/export | Samples were collected from a floating dock in the Scheldt estuary |
| Disturbance | No disturbance was caused to the habitat |

# Reporting for specific materials, systems and methods

We require information from authors about some types of materials, experimental systems and methods used in many studies. Here, indicate whether each material, system or method listed is relevant to your study. If you are not sure if a list item applies to your research, read the appropriate section before selecting a response.

### Materials & experimental systems

| n/a | Involved in the study | |
|---|---|---|
| ☒ | ☐ | Antibodies |
| ☐ | ☒ | Eukaryotic cell lines |
| ☒ | ☐ | Palaeontology and archaeology |
| ☒ | ☐ | Animals and other organisms |
| ☒ | ☐ | Clinical data |
| ☒ | ☐ | Dual use research of concern |
| ☒ | ☐ | Plants |

### Methods

| n/a | Involved in the study | |
|---|---|---|
| ☒ | ☐ | ChIP-seq |
| ☒ | ☐ | Flow cytometry |
| ☒ | ☐ | MRI-based neuroimaging |

## Eukaryotic cell lines

Policy information about cell lines and Sex and Gender in Research

| | |
|---|---|
| Cell line source(s) | For RT-qPCR, Cylindrotheca closterium cell lines A6 (DCG 0980, MT-) and MC4 (DCG 1267, MT+) were used, obtained from the Belgian Coordinated Collection of Micro-organisms (BCCM/DCG, https://bccm.belspo.be/about-DCG) |
| Authentication | Not relevant to diatom micro-algal cultures |
| Mycoplasma contamination | Not relevant to diatom micro-algal cultures |
| Commonly misidentified lines (See ICLAC register) | Not relevant to diatom micro-algal cultures |

## Plants

| | |
|---|---|
| Seed stocks | *Report on the source of all seed stocks or other plant material used. If applicable, state the seed stock centre and catalogue number. If plant specimens were collected from the field, describe the collection location, date and sampling procedures.* |
| Novel plant genotypes | *Describe the methods by which all novel plant genotypes were produced. This includes those generated by transgenic approaches, gene editing, chemical/radiation-based mutagenesis and hybridization. For transgenic lines, describe the transformation method, the number of independent lines analyzed and the generation upon which experiments were performed. For gene-edited lines, describe the editor used, the endogenous sequence targeted for editing, the targeting guide RNA sequence (if applicable) and how the editor was applied.* |
| Authentication | *Describe any authentication procedures for each seed stock used or novel genotype generated. Describe any experiments used to assess the effect of a mutation and, where applicable, how potential secondary effects (e.g. second site T-DNA insertions, mosiacism, off-target gene editing) were examined.* |
